# Spectroscopic Signatures of Hydrogen-Bonding Motifs in Protonic Ionic Liquid Systems: Insights from Diethylammonium Nitrate in the Solid State

**DOI:** 10.1021/acs.jpcc.1c05137

**Published:** 2021-10-27

**Authors:** Isabel Vázquez-Fernández, Kacper Drużbicki, Felix Fernandez-Alonso, Sanghamitra Mukhopadhyay, Peter Nockemann, Stewart F. Parker, Svemir Rudić, Simona-Maria Stana, John Tomkinson, Darius J. Yeadon, Kenneth R. Seddon, Natalia V. Plechkova

**Affiliations:** † The QUILL Research Centre, School of Chemistry and Chemical Engineering, The Queen’s University of Belfast, Belfast BT9 5AG, Northern Ireland, U.K.; ‡ Materials Physics Center, CSIC-UPV/EHU, Paseo Manuel de Lardizabal 5, Donostia-San Sebastian 20018, Spain; § Centre of Molecular and Macromolecular Studies, Polish Academy of Sciences, Sienkiewicza 112, Lodz 90-363, Poland; ∥ Donostia International Physics Center (DIPC), Paseo Manuel de Lardizabal 4, Donostia-San Sebastian 20018, Spain; ⊥ Department of Physics and Astronomy, University College London, Gower Street, London WC1E 6BT, U.K.; # Ikerbasque, Basque Foundation for Science, Plaza Euskadi 5, Bilbao 48009, Spain; ¶ ISIS Facility, Rutherford Appleton Laboratory, Chilton, Didcot OX11 0QX, U.K.; ∇ Department of Materials, Imperial College London, Exhibition Road, London SW7 2AZ, U.K.

## Abstract

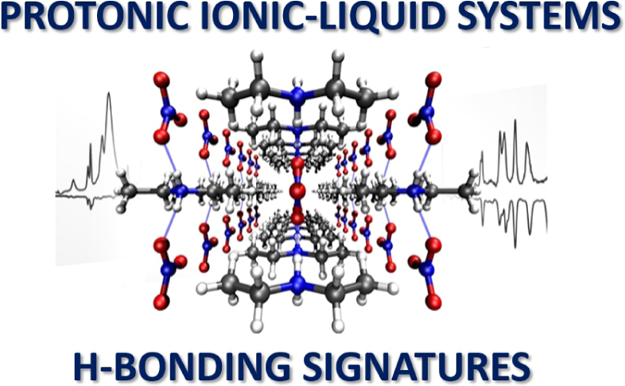

Diethylammonium nitrate, [N_0 0 2 2_][NO_3_], and its perdeuterated analogue, [N_*D D* 2 2_] [NO_3_], were structurally characterized and studied by infrared, Raman, and inelastic neutron scattering (INS) spectroscopy. Using these experimental data along with state-of-the-art computational materials modeling, we report unambiguous spectroscopic signatures of hydrogen-bonding interactions between the two counterions. An exhaustive assignment of the spectral features observed with each technique has been provided, and a number of distinct modes related to NH···O dynamics have been identified. We put a particular emphasis on a detailed interpretation of the high-resolution, broadband INS experiments. In particular, the INS data highlight the importance of conformational degrees of freedom within the alkyl chains, a ubiquitous feature of ionic liquid (IL) systems. These findings also enable an in-depth physicochemical understanding of protonic IL systems, a first and necessary step to the tailoring of hydrogen-bonding networks in this important class of materials.

## 1 Introduction

Ionic liquids (ILs) have attracted a phenomenal amount of attention over the past two decades,^1^ but the cynosure has been principally the development of industrial applications and sustainable processes.^2,3^ Although many theoretical models of IL structures have been developed and published, we are still a long way from predictive certainty, or even good qualitative models for describing the principle interionic interactions.^4^ As an illustration, many studies assume the presence of discrete ion pairs, even though neutron diffraction studies have clearly demonstrated that there is no evidence for their existence.^5^ The presence and nature of hydrogen bonding (H-bonding) in ILs was originally controversial, but now it is generally accepted.^6^ This work focuses on protonic ILs, and the search for spectroscopic signatures of H-bonds that have a significant impact on physicochemical properties. The term protonic ILs (PILs) is used to distinguish strongly acidic cations (i.e., those which contain readily dissociable protons) from protic ILs, which contain very weakly acidic protons.

As ILs consist only of anions and cations, it is generally assumed that Coulombic interactions dominate the properties of these compounds.^7^ However, the determination of H-bonded environments is critical for the understanding of how these interactions influence lattice energies, melting points, and the general behavior of ILs, for example, ref (8). It has been estimated that, for organic salts, the H-bond may contribute up to 25% of the lattice energy.^9^ Extended H-bonded networks in the liquid phase have been reported, with possible implications for both the structure and solvent properties of ILs.^6^ Bonhôte et al.^10^ found that H-bonding in ILs can have direct effects on physical properties. Using far-infrared (IR) spectroscopy, Ludwig and co-workers reported that there is an enhanced cation–anion interaction due to H-bonding in pure imidazolium ILs.^11^ The observed frequency shifts could be related to increased force constants, indicating stronger cation–anion interactions. Ab initio methods suggested a close relationship between the calculated interaction energies of IL aggregates and the measured intermolecular stretching frequencies.^11^ According to Hunt and co-workers, “doubly ionic” H-bonds occur when an H-bond forms between a cation and an anion,^6^ and are a key feature of ILs. The H-bonds in a protonic IL will always be stronger than cation–neutral or anion–neutral H-bonds, with neutral–neutral being the weakest. In line with the above, the primary objective of this work is to search for signatures of interionic H-bond motifs in PILs using state-of-the-art experimental and computational methods. To optimize our chances of success, we selected several series of ILs to study: the nitrate salts of alkylammonium, [N_0 0 0 *n*_]^+^, dialkylammonium, [N_0 0 *n n*_]^+^, and trialkylammonium, [N_0 *n n n*_]^+^, cations. Key to our strategy was the synthesis of the perdeuterated analogues, [N_*D D D n*_][NO_3_], [N_*D D n n*_][NO_3_], and [N_*D n n n*_][NO_3_], as the position of the D···O bands is expected to occur at significantly different frequencies than for H···O.^12^ As a first step in this program, this work specifically focuses on the diethylammonium nitrate salts [N_0 0 *2 2*_][NO_3_] and [N_*D D 2 2*_][NO_3_] (hereafter DEAN), for which we have a substantial corpus of structural, spectroscopic, and theoretical data.

In the previous searches for H-bonding signatures in ILs, Ludwig and co-workers measured and interpreted the IR spectra for systems analogous to ours: [N_0 0 0 2_][NO_3_], [N_0 0 0 3_][NO_3_], [N_0 0 1 1_][NO_3_], [N_0 1 1 1_][NO_3_], and [N_1 1 1 1_][NO_3_].^13,14^ Additionally, the interaction energies between anions and cations in triethylammonium triflate, bistriflamide, and methanesulfonate ILs were dissected from far-IR measurements of protonated and perdeuterated species.^15^ The phonon-like hydrogen-bond modes in molten PILs have also been discussed,^16^ clearly suggesting that collective vibrational motions persist above the melting point. This increased interest in low-energy vibrational excitations calls for the use of neutron scattering as the natural method of choice. However, to date, reports on the application of neutron spectroscopy beyond the μeV range to study ILs are extremely sparse, and have been primarily devoted to the study of the so-called boson peak.^17,18^ The technique of our choice here is (incoherent) inelastic neutron scattering (INS), which unlike the complementary techniques of IR and Raman spectroscopy exhibits no optical selection rules, thus providing direct access to nuclear motions. Particularly, we take advantage of its exquisite sensitivity to proton dynamics in the region below 1000 cm^–1^.^19,20^ For the first time, we report an exhaustive, computationally supported study of PILs using INS spectroscopy, identifying distinct modes associated with cation–anion interactions. We further illustrate the ability of our joint experimental and computational efforts to elucidate structural peculiarities in the crystal structures induced by the conformational freedom of the constituting molecules.

## 2 Methods

### 2.1 Synthesis

Aqueous nitric acid (5 M, 1.0 mol equiv) was added dropwise to neat diethylamine (1.1 mol equiv; 99%, Ex Aldrich) and cooled to −78 °C in a round-bottomed flask. The reaction is exothermic and pressure builds up after each acid addition. After all the acid was added, the mixture was left to warm up to room temperature (2 h), and then stirred for a further hour. The water and diethylamine excess were removed by freeze-drying at 0.03 mbar pressure for three cycles. The resulting product is a pale yellow hygroscopic powder, with a water content lower than 300 ppm (determined by Karl Fischer titration). C_4_H_12_N_2_O_3_: found (theory); C, 35.66 (35.29); 8.96 (8.88); and 20.29 (20.58)%. [N_*D D* 2 2_][NO_3_] was prepared from [N_0 0 2 2_][NO_3_] by heating in D_2_O at 40 °C overnight and subsequent evaporation of the solvent by freeze drying. The process was repeated until there were no N–H bands detected by IR spectroscopy (typically 4–5 times).

### 2.2 Thermal Analysis

Differential scanning calorimetry (DSC) scans were recorded at 5 °C/min from −90 to 200 °C (standard heating and cooling ramp over five cycles), using a TA DSC Q2000 Instrument, with a refrigerating cooling system RCS 90. Dry dinitrogen gas was purged through the DSC cell with a flow rate of ca. 20 cm^3^/min. Due to its hygroscopic character, the sample was prepared in a glovebox, using hermetic platinum pans to avoid any contact with air.

Optical textures were observed with an Olympus BX50 polarizing microscope equipped with a LINKAM TH600 hot-stage, and a TP92 programmable temperature controller. The sample was sandwiched between two thin plastic microscope slides and then placed on the controlled hot-stage. The hot-stage was mounted on the working stage of the microscope and heated at 5 °C/min.

### 2.3 Crystallographic Study

Single-crystal X-ray diffraction (SXRD) measurements for [N_0 0 2 2_][NO_3_] were performed on a Rigaku Supernova Dual Source SXRD diffractometer, equipped with a nitrogen cryostat. The measurements were carried out at 120 K, using a Cu–Kα source (λ = 1.54184 Å). The images were interpreted and integrated with the program CrysAlisPro (Agilent Technologies). The structure was solved with Olex2,^21^ by employing direct methods as implemented in the ShelXS structure solution program, and refined by full-matrix least-squares on *F*^2^ with the ShelXL program package. Non-hydrogen atoms were refined anisotropically, whereas the hydrogens have been refined in the riding mode on their carrier atoms. The isotropic temperature factors for hydrogens were fixed at 1.2 times *U*_(eq)_ of the parent atoms (for methyl and hydroxyl groups, the factors were fixed at 1.2 × *U*_(eq)_). In order to measure the powder XRD (PXRD) patterns (low-resolution), the powder sample was sealed under an argon atmosphere in quartz capillaries, and subsequently measured at 120 and 298 K.

High-resolution PXRD data were collected at ambient temperature using a PANalytical X’Pert PRO powder diffractometer, equipped with the same X-ray source (λ = 1.54184 Å). X-rays were generated from a Cu anode supplied with 40 kV and a current of 40 mA. Data were recorded for 2Θ between 5 and 90°, in steps of 0.017° per 5 s.

The intermolecular contacts were identified by calculating the reduced (promolecular) density gradient (RDG)^22^ in the localized regions of electron density within the crystal voids, as well as using Hirshfeld surface analysis.^23^ The calculations were performed with the help of the NCIPLOT^24^ and CrystalExplorer^25^ programs, respectively.

### 2.4 Optical Spectroscopy

Attenuated total reflection–Fourier transform infrared (ATR–FTIR) spectra were collected with a PerkinElmer Spectrum 100 FT-IR spectrometer, using a Universal ATR Sampling Accessory (DiCompTM diamond coated zinc-selenide crystal). Data were recorded at room temperature, in the range of 4000–400 cm^–1^, with a resolution of 4 cm^–1^. The samples were analyzed by placing a small amount directly onto the ATR accessory, with N_2_ gas flowing over it via a glass conical funnel attached to the dinitrogen gas tube. The funnel was kept as close as possible to the sample, to minimize exposure to moisture.

Raman spectra were collected using a Perkin Elmer RamanStation 400 F spectrometer, equipped with an Echelle spectrograph and a CCD detector. A 785 nm laser line was used, with a spectral range of 3000–95 cm^–1^. The samples were analyzed using sealed glass cuvettes.

### 2.5 Inelastic Neutron Scattering

The INS spectra were recorded using the high-resolution (Δ*E*/*E* ∼ 1.25%) broadband spectrometer TOSCA optimized for vibrational spectroscopy in the 0–4000 cm^−1^ region, operating at the ISIS Pulsed Neutron and Muon Source of the Rutherford Appleton Laboratory (Chilton, UK).^26−30^ The samples (ca. 2–3 g) were placed in sealed flat thin-walled aluminum cans (which filled the neutron beam at the sample position) mounted perpendicular to the incident neutron beam using a standard TOSCA center stick. Sample preparation was performed at 1 bar in an inert-gas atmosphere, to avoid exposure to water vapor. To reduce the impact of the Debye–Waller factor (DWF) on the observed spectral intensities, the samples were cooled down to approximately 10 K with a closed-cycle refrigerator, and the spectra were recorded for a few hours. The Mantid software package was used to reduce and analyze the experimental data.^31^

### 2.6 Ab Initio Modeling

Theoretical calculations were performed under periodic boundary conditions using the low-temperature crystallographic data as a starting model. The plane-wave pseudopotential (PW-PP) formulation of density functional theory (DFT) was employed as implemented in CASTEP.^32,33^ In brief, the Perdew–Burke–Ernzerhof (PBE) functional within the generalized-gradient-approximation (GGA) was used throughout this study.^34^ The core electrons were described by a set of hard, norm-conserving PPs (NCPPs), while the electronic wave functions were defined using a PW basis set with a kinetic energy cutoff of 1050 eV, converging the self-consistent field (SCF) to within 1 × 10^–12^ eV/atom. A Monkhorst–Pack grid was used to maintain a constant *k*-spacing of 0.05 Å^–1^. The maximum *G*-vector of the fast Fourier transform grid, *G*_max_, was defined as three-quarters of the ideal grid size, and the fine-grid multiplier was set to four.

A fixed-cell methodology has been applied throughout this work. All internal coordinates were accurately relaxed to minimize residual atomic forces. The convergence criteria in the variation of the Hellmann–Feynman forces and the maximum displacement were defined as 1 × 10^–5^ eV/Å and 1 × 10^–6^ Å, respectively. Phonon properties were explored using Harmonic lattice dynamics (HLD) using a reciprocal-space implementation of density functional perturbation theory (DFPT).^35−37^ The linear-response approach was also used to calculate the IR and Raman activities, the latter transformed into the intensities by accounting for the laser excitation line and the temperature conditions. The same numerical methodology was further applied to the more extended models considered in this work, unless otherwise stated. Isotopic substitution was implemented at the level of dynamical-matrix diagonalization, therefore only affecting the effective mass of the same equilibrium model at 0 K. The resulting phonon eigenvalues and eigenvectors were used for the simulation of the TOSCA spectra.^38,39^ Isotopic substitution was accounted for via the use of the appropriate neutron-scattering cross section for each isotope.

In addition to the above, we have also performed a series of ab initio molecular dynamics (AIMD) simulations for a primitive cell model, to map the influence of anharmonicity on the vibrational density of states (VDoS). In this case, Born–Oppenheimer MD (BOMD) has been applied with a 0.5 fs time step, using exactly the same numerical settings, apart from the SCF convergence, which has been reduced to 2.5 × 10^–7^ eV/atom. Initially, for each target temperature, a 5 ps equilibration run was performed in the canonical ensemble (*NVT*) using a Nose–Hoover thermostat. Subsequent production runs of 25 ps were collected in the microcanonical ensemble (*NVE*), serving as the input for the modeling of hydrogen-projected VDoSs via the explicit calculation of the velocity autocorrelation function (VACF). The analysis and postprocessing of the BOMD production runs were performed with the MDANSE code.^40^

## 3 Results and Discussion

**Figure 1:**
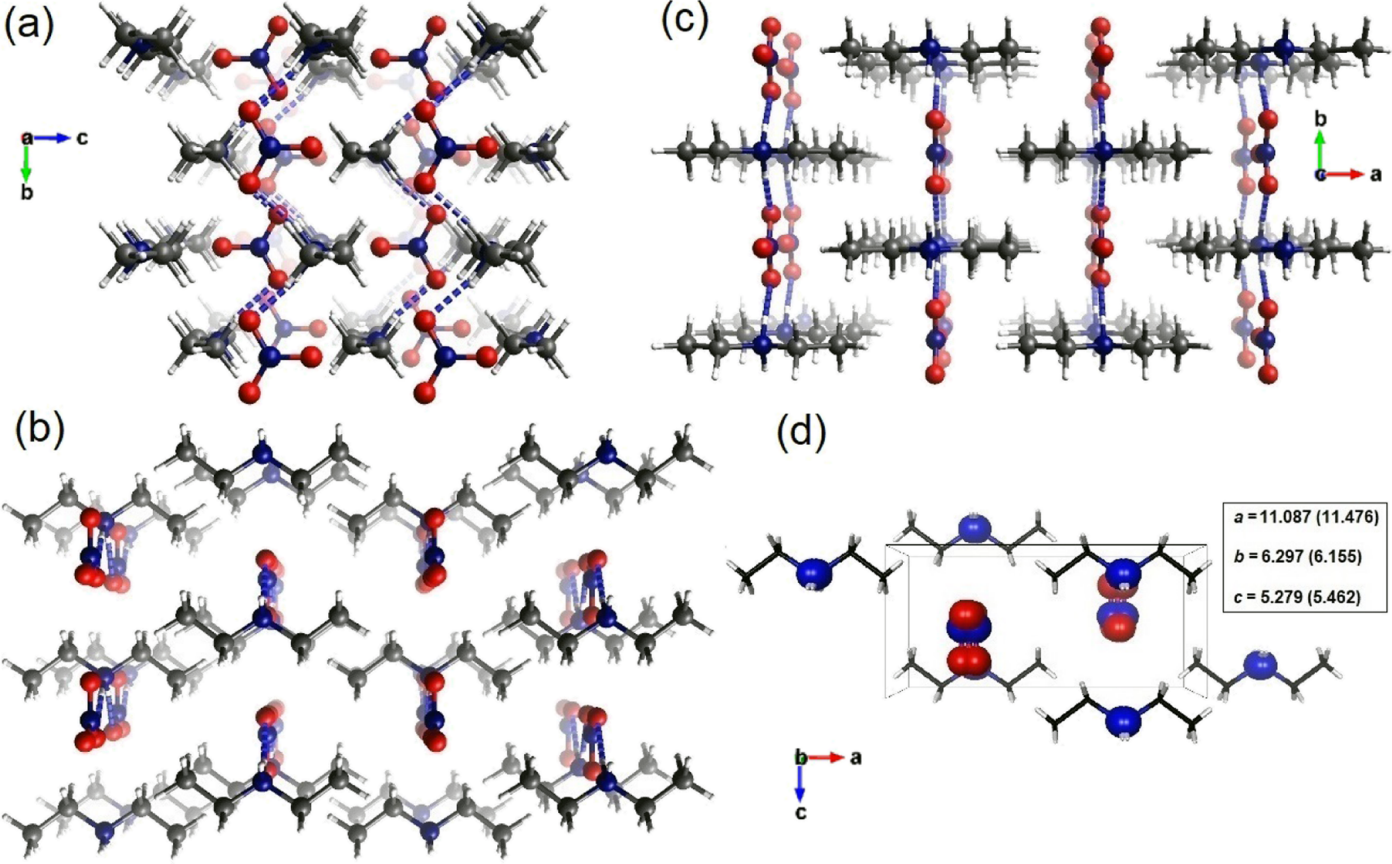
(a–c) The crystal packing of the diethylammonium nitrate crystal [N_0 0 2 2_][NO_3_] (orthorhombic, *Pmmn*), according to low-temperature (120 K) SXRD measurements, shown in alternative 2 × 2 × 2 supercell projections (along the *a*, *b*, and *c* crystallographic axes, respectively), with the (putative) hydrogen bonds marked as dashed blue lines (with O···H distances below 2 Å). (d) Projection of the unit-cell along the *b*-axis according to the low-temperature SXRD measurements. The heavy atoms are shown as spheres and are superimposed with the PXRD refinements (298 K) to illustrate cell expansion with temperature. The cell constants at both temperatures are shown in Å in the inset (room-temperature values are given in parentheses).

The crystallographic structure of [N_0 0 2 2_][NO_3_] is presented in Figure 1 (see the Supporting Information, for the atomic coordinates deposited as a CIF file). The structure is orthorhombic, with the *Pmmn* space group, with half of each counterion in the asymmetric unit. The nitrate ([NO_3_]^−^) and diethylammonium ([Et_2_NH_2_]^+^) counterions are aligned in the *bc*-mirror-plane propagating the interactions between the counterions to infinity along the *b*-axis (see Figure 1a–c). The result of this structural arrangement is the possibility of forming a H-bonded network, where all the H-donors and H-acceptors would be occupied. This picture is supported by the analysis of both the non-covalent interaction (NCI)^22^ index and the Hirshfeld surface^41^ presented in the Supporting Information (see Figures S1 and S2), showing signatures of the formation of well-defined H-bonding at each site of the [Et_2_NH_2_]^+^ cation with *C*_2*v*_ symmetry. According to the SXRD structure, the interactions with the cations lower the site-group symmetry of the anion from *D*_3*h*_ to *C*_2*h*_, where the two N–O bonds involved in stronger interactions with hydrogens have a longer distance (*d*N–O = 1.250 Å) than the one involved in the weaker interaction (*d*N–O = 1.240 Å). Similarly, the major and minor distances between the heavy atoms (*d*N···O) equal to 2.867 and 3.151 Å, respectively. Standard structural criteria would classify the stronger interaction as an ionic H-bond of moderate strength.^6^ The NCI analysis does not support an H-bond character for the weaker interaction. This is further corroborated by the analysis of the AIMD trajectories, scrutinizing the evolution of the local structure as a function of temperature. As shown in Figures S3 and S4 of the Supporting Information, the [NO_3_]^−^ ions manifest a high propensity for both in-plane and out-of-plane motions with increasing temperature, indicating a poor ability of the weaker interaction to stabilize its geometry. This is corroborated by the analysis of the thermal motions in the SXRD data (see Figure S2), showing pronounced out-of-plane displacements of the weakly bound oxygen atom, and thus ruling out the formation of a three-centered hydrogen bond with one hydrogen donor.

The synthesized [N_0 0 2 2_][NO_3_] powder has been characterized by DSC and PXRD. DSC reveals a solid–solid phase transition occurring upon heating to 60 °C, characterized by an enthalpy of 7.8 kJ/mol (see Figure S5). The presence of a solid–solid phase transition has been earlier detected by Mangialardo et al. at 55 °C with temperature-dependent Raman measurements,^42^ which to the best of our knowledge is the only spectroscopic study of DEAN reported to date. The melting transition has been found at 101 °C, with an associated enthalpy of 6.7 kJ/mol. However, most importantly, no signatures of a low-temperature structural phase transition can be observed.

**Figure 2:**
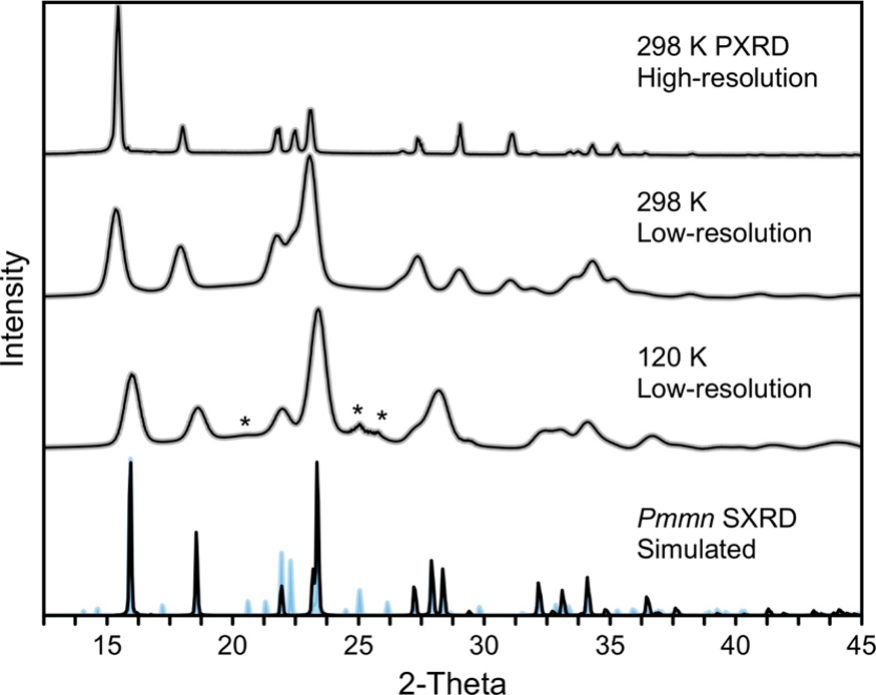
Powder diffraction patterns of [N_0 0 2 2_][NO_3_]. Low-resolution patterns were recorded using a SXRD instrument. The high-resolution diffractogram was recorded at room temperature using a standard setup. The bottom panel shows the simulated diffractogram from the single-crystal structure solved at 120 K. The asterisks in the low-temperature diffraction data suggest the presence of a fraction of molecules with the gauche conformation. This finding has been confirmed by model simulations assuming a 25% contribution from the alternative conformers in the lattice (blue patterns in the bottom panel; see further discussion in the main text).

Figure 2 shows the PXRD patterns recorded at both low temperature (120 K) and under ambient conditions. The comparison between both patterns shows their continuous evolution with temperature, confirming no structural phase transition. The calculated diffraction patterns shown in the bottom panel confirm that the structure of the powder specimen is the same as for the single crystal, following the intensity distribution and exact positions of the Bragg peaks. However, some reflections observed experimentally for a powder specimen are absent in the calculated patterns. Because the cell volume remains the same for both powder and single crystals, we exclude the scenario whereby intercalation of other species such as water has taken place. Instead, we attribute the aforementioned differences to a fraction of cations deviating from the site-symmetry defined by the all-trans conformation while preserving the long-range ordering revealed with SXRD. To support this finding, we compare the simulated patterns with alternative ones (see the light-blue features), which were calculated from a model including 25% of cations showing the gauche conformation (discussed in more detail below). The propensity of the [Et_2_NH_2_]^+^ cations to conformational changes is also supported by the AIMD simulations, as illustrated in Figure S6.

The interactions between the counterions determine a packing conformation with the alternating layers. The non-polar domains are formed by staggered alkyl chains, whereas the polar domains are intercalated in between, forming alternating layers. The NCI analysis (see Figure S1) highlights the importance of the presence of non-specific stabilizing interactions of van der Waals (vdW) nature, largely spread over the whole cell volume. The high-resolution PXRD patterns have also been collected at room temperature, providing additional structural data under ambient conditions. The cell constants have been extracted assuming the orthorhombic *Pmmn* space group. These data are shown in Figure 1d, along with the heavy-atom projections associated with cell expansion. The clear temperature dependence can be linked to a pronounced shearing along the long crystal-axis direction. This can be explained by the presence of considerable voids in the crystal lattice, where packing along the *a*-axis is primarily stabilized by weak vdW interactions between the counterions (see Figures S1 and S2). The weak nature of the aforementioned interactions is responsible for a marked thermal expansion, and can also promote conformational changes within the site-chains, an ubiquitous feature of IL systems.^43,44^

The studied material has been subjected to complementary vibrational spectroscopy analysis. The vast majority of research on ILs employing IR and Raman spectroscopy rely on the use of few-atom models involving single molecules or their clusters, and confronting the calculations with the experimental results in the molten state.^45−49^ This is often dictated by the aforementioned lack of crystallographic data, and so relies on a poorly defined molecular environment. Another obstacle is the dynamical smearing of the vibrational features recorded experimentally, where the discrete structure of the spectral bands tends to be lost. These challenges were recognized by Kirchner and co-workers, resulting in the development of new methodologies for accurate predictions of the vibrational spectra of molten ILs via the use of AIMD simulations.^50−53^ Further developments on this front can account for statistical averaging in complex molten-salt environments, as recently illustrated in ref (54). In the present work, we follow an alternative strategy by capitalizing from our knowledge of the crystal environment and provide a comprehensive multispectral analysis. To the best of our knowledge, this is also the first complementary optical and neutron spectroscopy study of ILs reported to date, heavily underpinned by solid-state DFT calculations.

**Figure 3:**
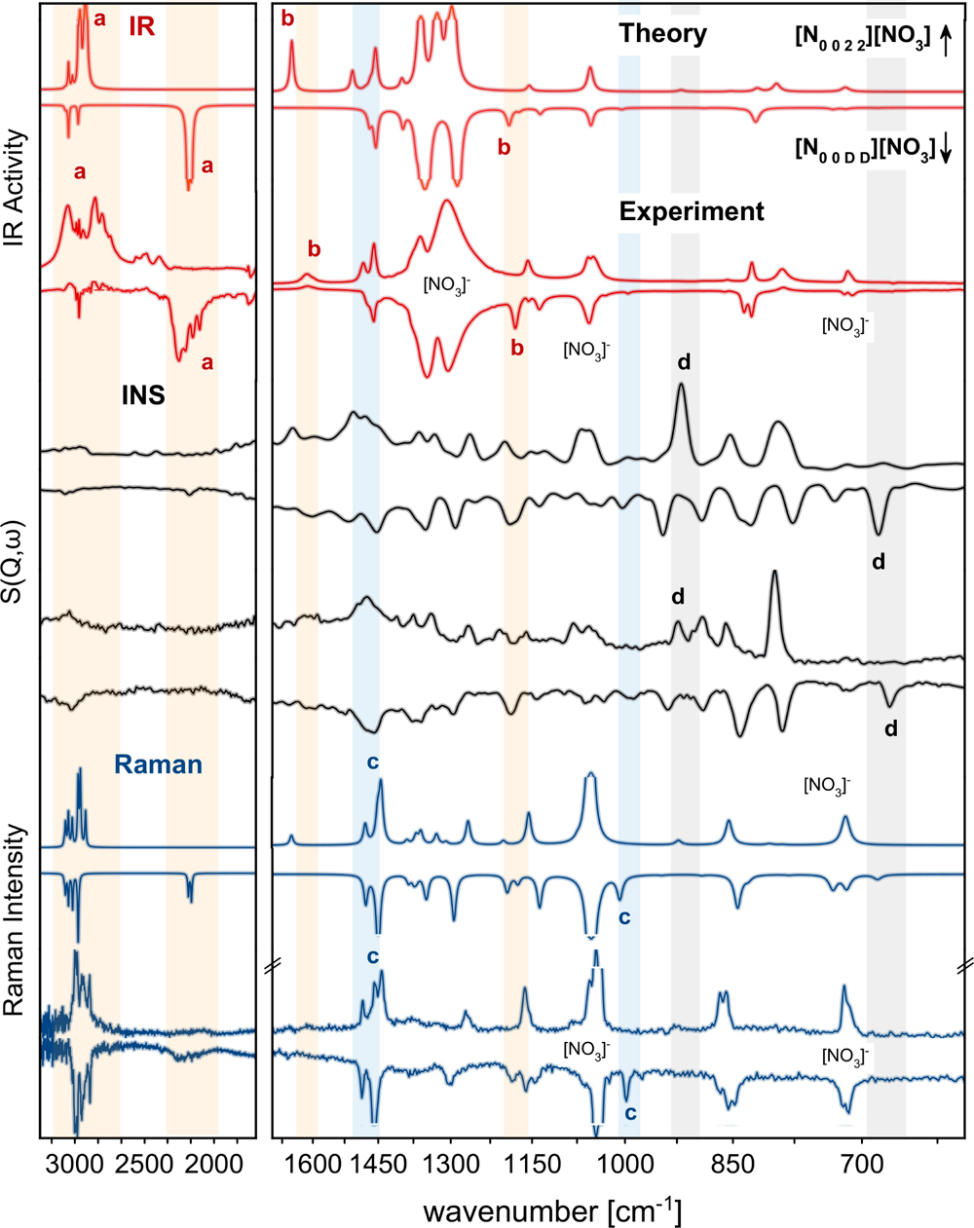
Experimental and theoretical (harmonic lattice dynamics, PBE/1050 eV/hard-NCPPs) IR, INS, and Raman spectra of hydrogenous (↑) [N_0 0 2 2_][NO_3_] and perdeuterated (↓) [N_0 0 *D D*_][NO_3_] diethylammonium nitrate crystals in the high- (3250–1700 cm^–1^) and intermediate-energy (1700–600 cm^–1^ on the log scale) range. The characteristic N–H···O vibrations are labeled as (a–d) and highlighted with vertical shaded bars, color-coded according to the spectroscopic technique most sensitive to a given spectral feature (red for IR; grey for INS; and blue for Raman).

The experimental spectra obtained for both protonated and perdeuterated samples in the intermediate-energy range (>600 cm^–1^) are shown in Figure 3. This figure illustrates the complementarity of the spectroscopic techniques, each bringing their own benefits and disadvantages. IR spectroscopy is superbly sensitive to charge fluctuations, thus leading to intense features sensitive to H-bonding. However, the long-range coupling associated with the excitation of polar phonons (ungerade symmetry) in the IR spectrum is expected to affect the dielectric response of the material considerably, resulting in both frequency shifts and intensity modulations and spectral broadening.^55,56^ Because the crystallographic model of this ionic system is defined by a centrosymmetric space group (*Pmmn*), the Raman spectrum will correspond to the non-polar transverse optical phonons (gerade symmetry), offering much better resolved spectra. However, because the polarizability for an atom normally grows with the number of electrons, it is hardly sensitive to hydrogen dynamics, and the features under interest suffer from a low intensity. As relying on the dipole approximation, IR and Raman are both subjected to optical selection rules, with a mutual-exclusion rule applying to centrosymmetric systems. On the contrary, for closed-shell systems exhibiting no unpaired electron density, the interaction probed with INS is with the nuclei rather than with the electron cloud, and the optical selection rules no longer apply. Moreover, because the neutron has a mass commensurate with those of atomic species, an inelastic collision with a nucleus involves a significant transfer of both momentum, *Q* (Å^–1^), and energy *E*. Equation 1 gives the intensity of each vibrational transition at an energy transfer *E*_*i*_ = ℏω_*i*_, defined by the so-called scattering law *S*(*Q*,ω_*i*_)^57,58^ as follows
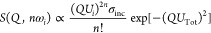
1where *n* is the quantum number of the *i*-th mode (*n* = 1 for a fundamental; *n* > 1, 2, 3, and so forth, for overtones and combination bands); and *Q* (Å^–1^) denotes the momentum transfer. The exponential term is known as the DWF. *U*_Tot_ stands for the total root-mean-square displacement of all the atoms in all (both internal and external) modes, and its magnitude is partially determined by the thermal motion of the molecule. The DWF results in the damping of the observed intensity, and to reduce its impact on the observed intensity, the samples are cooled to cryogenic temperatures.^26^ σ (barn) is the neutron-scattering cross section, which is an isotope-specific property. INS is superbly sensitive to hydrogen motions due to the high incoherent scattering cross section of this nuclide, which is an order of magnitude larger than any other nucleus at thermal neutron energies. Differences with deuterium are of particular significance, as spectral intensities undergo a significant suppression upon deuteration. The importance of the sensitivity of INS to hydrogen in the present case is further strengthened by the Hirshfeld surface analysis^23^ presented in Figure S2, estimating that H···H interactions contribute to more than 80% of the total number of close-contacts.

The direct access to nuclear dynamics offered by INS also has a great advantage for spectral modeling, where information on atomic displacements and the frequency associated with a given mode is generally sufficient to predict INS intensities (see, e.g., refs (38) and (59)), without any reference to atomic charges or the electron density around the nucleus. For well-defined crystalline solids, HLD calculations employing accurate potentials from electronic-structure calculations constitute a robust means of interpreting the low-temperature experimental data. However, an accurate description of molecular systems involving a considerable degree of charge transfer is a well-recognized challenge for DFT.^60−62^ This limitation arises from the use of semilocal approximations of the exchange–correlation (XC) functional leading to significant self-interaction errors (SIEs).^63,64^ The SIE plays a central role in electronic polarization, and generally leads to the overstabilization of interacting molecular species.^64^ The use of hybrid functionals, including a portion of exact Hartree–Fock (HF) exchange, is known to suppress the SIE,^65^ which, however, is a remedy still too expensive for its routine use in solid-state calculations. On the other hand, an accurate description of crystal packing requires the ability to account for dispersion forces,^56,66,67^ which conventional DFT does not capture, thus resulting in the destabilization of crystal environments.

To mitigate the above difficulties, as well as to avoid the arbitrariness and deficiencies of semiempirical vdW corrections, we have adopted a fixed-cell methodology, using the cell parameters measured with SXRD at 120 K. We have shown earlier a high robustness and versatility of such an approach in combination with standard (PBE) and hard-GGA (rPBE) GGA-type XC functionals for the prediction of the vibrational spectra. The adequacy of the approach has been confirmed for a number of diversely bonded, flexible, and complex molecular as well as ionic systems, studied over a broad frequency range and compared to both optical and INS data.^56,68−72^ This is also corroborated by our previous experience in the modeling of solids containing nitrate ions^55,73^ and nitro moieties.^74,75^ The adequacy of the adopted methodology in our particular case is examined in Figures S7 and S8, showing a zone-center mechanical stability of the *Pmmn* model. The reliability of our computational methodology is confirmed further in Figure 3, presenting theoretical spectra at intermediate energies calculated for both [N_0 0 2 2_][NO_3_] and [N_0 0 *D D*_][NO_3_] specimens. By visual inspection of Figure 3, we find an excellent agreement in terms of both band positions and intensity distributions for both specimens. A complete assignment of all experimental bands is provided in Table S1 and is supplemented with a collection of normal-mode animations associated with each vibration of interest. A quantitative analysis of the correlation between experimental and predicted vibrational energies is further provided in Figure S9, showing that most of the vibrational transitions are accounted for with an absolute error below 10 cm^–1^.

The spectral features highlighted in Figure 3 and marked as (a–d) were found to be particularly sensitive to isotopic substitution, thereby offering a means to identify those vibrational modes directly affected by cation–anion interactions. Furthermore, deuteration has a profound effect beyond these few bands, indicating that the internal modes contributing to the intermediate-energy range are strongly mixed in terms of the underlying normal-mode coordinates. Figure 3 supports this picture, illustrating that theoretical calculations become an indispensable tool to attain robust spectral assignments upon deuteration.

The first unambiguous signature of H-bond formation is provided by an analysis of the stretch region. By inspection of both Figure 3 and the quantitative analysis given in Figure S9, we note that the high-energy range cannot be described properly within the harmonic approximation, as it involves large-amplitude ν[N–H] vibrations [marked as (a)]. However, systematic deviations of semilocal DFT in the description of the potential energy surface for the stretching coordinates results in a fortuitous cancellation of errors,^76^ providing a reasonable match to the experimental data. This can be associated with a general overbinding tendency of PBE due to the aforementioned SIE deficiency,^77^ which may be particularly severe for the accurate prediction of H-bond energies.^78−80^

The stretching modes of the [NH_2_] and [ND_2_] fragments are easily identified in the IR spectra presented in Figure 3. A quantitative analysis of the red shift would require a reference, which, however, cannot be provided because there is no dialkylammonium solid without directional bonding that could be used to this end. However, one can still provide some evidence for H-bond formation based on the analysis of the spectral profiles. Figure 3 convincingly shows that these modes contribute with very high intensity to the IR spectrum, yet they are hardly detectable by Raman or INS. In addition to the red shift of the X–H stretch frequency, a well-known signature of H-bond formation is an induced dipole moment resulting in a strong increase in the absorption intensity of the IR band.^81^ Further evidence can be provided based on band-shape analysis. As shown in Figure 3, only two transitions are predicted with the harmonic-approximation, corresponding to the symmetric and antisymmetric ν[N–H] stretch. However, the experimental spectrum shows a much-broadened feature spanning over a few hundreds of reciprocal centimeters. This broadening is a result of the mode coupling involving the ν[N–H] coordinate. It has been recognized that anharmonicity gives rise to the coupling of the ν(X–H) vibrations with the low-energy modes, mainly involving the bridge stretching.^82^ The theory from Marechal and Witkowski^83,84^ further developed by Wójcik and Witkowski tackles this situation by introducing Fermi-resonance effects.^85,86^ Such a coupling leads to a quantized substructure of the ν(X–H) bands and a large broadening due to a quasi Franck–Condon progression, which is confirmed here with IR. The vibrational substructure is equally separated here by ca. 50 cm^–1^. The related IR absorption bands result from a direct coupling to the low-energy [NO_3_]^−^ modes or from the relaxation of the excited vibrational levels, which interact further with the lattice phonons. A similar substructure of this band for both hydrogenous and perdeuterated specimens suggests that deuteration hardly affects the external modes, a result that is confirmed in our discussion below. The band broadening induced by anharmonicity and associated mode-coupling makes these features hardly detectable by INS and Raman, as evidenced by a comparison between the experimental and theoretical spectra. In the case of INS, this comparison is further hampered by the characteristics of the broadband spectrometer used in the experiment.^87^

The use of complementary spectroscopic techniques along with deuteration also allows for a clear assignment of other vibrations involving the motion of hydrogen-bonded [NH_2_] motifs. The in-plane bending modes δ[NH···O] at ca. 1600 cm^–1^ [marked as (b) in the figures] are easily identified with IR^88^ and INS spectroscopy. The assignments have been further confirmed by the simulations (see Table S1 in the Supporting Information), showing a pronounced isotope effect,^12^ red-shifting the associated frequencies by nearly 400 cm^–1^. The coupling of the γ[NH_2_] vibrational coordinate to the skeleton stretching modes allows us to probe the related mode with Raman [marked as (c) in the figures], which also shows a significant frequency softening upon deuteration. INS spectroscopy was further found to be sensitive to the ρ[NH_2_] vibrations at around 920 cm^–1^, and these clearly red-shifted down to ca. 675 cm^–1^ upon deuteration [marked as (d)].

The frequency of the above-described modes (a–d) has been reasonably described by the HLD calculations in spite of the inherent errors of PBE. We nonetheless note considerable signatures of anharmonicity resulting in significant deviations in the predicted vibrational energies (see Figure S9). To explore these effects further, we have performed a series of classically thermostatted AIMD simulations at several temperatures. The production-run trajectories served as the input for the calculation of the hydrogen-projected VDoS via the explicit calculation of the VACF. The VDoS for a particular hydrogen atom *j* is given by the Fourier-transform of the VACF according to
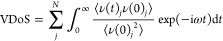
2where ν(*t*)_*j*_ is the velocity at time *t*.

**Figure 4:**
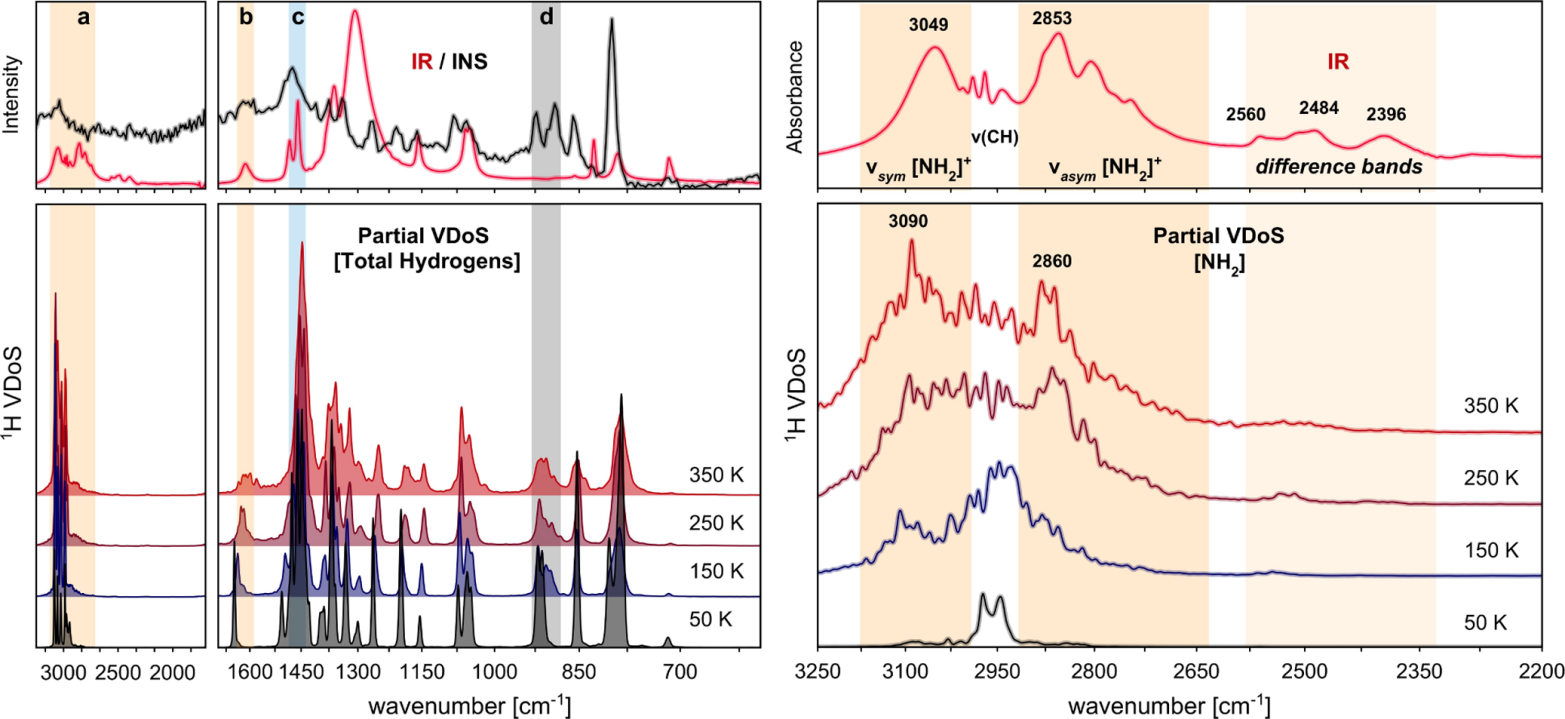
Hydrogen-projected VDoSs of hydrogenous [N_0 0 2 2_][NO_3_] obtained from ab initio MD simulations (PBE/1050 eV/hard-NCPP) in the microcanonical ensemble at selected temperatures. Left: the top panel shows the experimental IR and INS spectra. The bottom panel presents total contributions from hydrogens to the VDoS. The characteristic N–H···O vibrations are labeled as (a–d) and highlighted with vertical shaded bars, as in previous figures. Right: the top panel shows the experimental IR spectrum in the high-energy range. The bottom panel presents hydrogen-projected partial VDoSs for the [NH_2_] moiety.

The resulting hydrogen-projected VDoSs are shown in Figure 4. Inspection of the figure (see the left panel) further confirms our previous assignment of the main H-bonding signatures. However, due to the presence of vibrations associated with CH_2_CH_3_ hydrogens (85% of the total), NH···O modes are hardly discernible at first sight. By analyzing the temperature-dependence of the respective bands, one can, however, easily discern these NH···O modes, as they are characterized by a pronounced frequency-softening. The presence of a softened potential confirms that hydrogen is under the strong influence of the N and O atoms across the hydrogen bridge. At this point, we note difficulties in describing the zero-point motion in classical AIMD, requiring the use of an effective temperature. Hence, anharmonicity cannot be properly accounted at low temperatures because only the harmonic part of the potential well is significantly sampled.^89^ Introducing an effective temperature in classical simulations (which is, however, a well-recognized problem, see ref (90)) allows correcting the theoretically predicted frequencies for mechanical anharmonicity when referring to the highest classical temperature (350 K) used in the simulations [see the band marked as (a–d)].

As clearly seen from the calculated VDoSs compared to the INS spectrum, a pronounced intensity of the ν[N–H] band observed in IR stems from large charge fluctuations induced by H-bond formation, which otherwise could not be detected due to the prevalent number of CH_2_CH_3_ protons (see the INS spectrum for comparison, emphasizing the ν[CH_2_] and ν[CH_3_] modes). At this stage, we note that a detailed interpretation of the IR spectrum in the ν[CH] regime is beyond the capabilities of the adopted methodology, being affected by Fermi resonances along with combinations and overtones prohibiting a straightforward assignment.^91−93^ A high density of vibrational states for hydrogen atoms associated with the alkyl fragments also clarifies the presence of the hump in the INS spectrum over ca. 1500–1300 cm^–1^. Clear signatures of anharmonicity can be also deduced from the temperature dependence of the VDoS around 900 cm^–1^, associated with ρ[NH_2_] vibrations. The soft potential-energy landscape associated with these modes resolves the apparent discrepancies between the experimental INS intensities of these modes and those predicted with HLD.

The right panel of Figure 4 further scrutinizes the partial contributions from the [NH_2_] fragments to the high-energy VDoS (see Figure S10 for further insights at lower energies). The figure clearly illustrates the failure of harmonic approximation, red-shifting and blue-shifting the symmetric and antisymmetric ν[NH_2_] stretching frequencies by ca. 90 and 70 cm^–1^, respectively. As evidenced by the figures, accounting for mechanical anharmonicity cancels out these errors to a great extent, providing very good reproduction of the spectral profile [the same applies to other characteristic H-bond vibrations shown in Figure S10, marked as (b) and (c)]. This has more general implications in the understanding of IR response of secondary amine salts because the vibrational assignments of the ν[NH_2_] stretching frequencies in the literature remain vague and largely inconsistent to date.^94^ Our calculations rule out the assignment of the ν[NH_2_] modes to the features commonly occurring between approximately 2500 and 2400 cm^–1^ in the IR spectra.^94^Figure 4 shows convincingly that these bands stem from anharmonicity, most likely corresponding to difference bands captured by the simulations at the higher temperatures, where excitations of low-energy phonons also play a role.

At this stage, we address the question of how severe is the overbinding effect of PBE in the description of the hydrogen bridges in DEAN. To this end, the potential-energy well of the hydrogen atom in a single N···O bridge has been calculated by displacing the atom from its equilibrium position (see Figure S11). The calculated potential energy profile is characteristic of moderate-strength H-bonds,^82^ as evidenced by the absence of the double-well seen for strong interactions. The potential wells were calculated using several models (see Figure S11), including a conformationally disordered [Et_2_NH_2_]^+^ cation. Of relevance to future research of the molten phases, the calculations show highly directional H-bonds, being hardly affected by conformational disorder. The vibrational eigenvalues associated with this well have been calculated via a numerical solution of the one-dimensional Schrödinger equation,^95^ assuming the mass of the oscillator to be that of the proton. The predicted 0 → 1 transition is estimated at ca. 2700 cm^–1^, below the experimentally observed position of the ν[NH_2_] band (ca. 2850 cm^–1^). A very good reproduction of the antisymmetric stretching band by AIMD (ν[NH_2_] = ca. 2850 cm^–1^) using an effective temperature of 350 K signals the importance of nuclear quantum effects (NQEs) and associated zero-point energies, which are not included in the classical simulations from the outset, and are expected to further drag the predicted band position to lower energies if accounted for explicitly in the simulations.^96,97^ The presented analysis also rules out the propensity of the ν[N–H] stretch vibration to drive proton migration in the low-temperature phase of DEAN, which would not be accessible at room temperature. Consequently, and of relevance to the interpretation of the experimental spectra, these findings do not support the coexistence of nitric acid^98^ and ethylamine in the crystal lattice of DEAN.

Further conclusions on the formation of H-bonding in DEAN can be also inferred from the analysis of [NO_3_]^−^ vibrations. As mentioned above, the nitrate anion has lowered its symmetry from *D*_3*h*_ in the molecular form to *C*_2*h*_ in the solid state.^99−102^ The lowering of the local symmetry down to *C*_2*h*_ results in an increase of the number of IR-active bands from three to six. Nevertheless, not all the resulting bands can be clearly observed due to their low intensity and spectral congestion. Deuteration slightly affects the frequencies of the identified [NO_3_]^−^ bands, for instance, with a 15 cm^–1^ red shift of the ν_asymm_[NO_3_]^−^ mode around 1350 cm^–1^. The sensitivity of the [NO_3_]^−^ ions to isotopic substitution can also be considered as strong evidence for H-bond formation, indicating coupling in the dynamical matrix between the light and heavy species involved. Interestingly, isotopic substitution also affects the IR intensity of [NO_3_]^−^ modes, which suggests a considerable charge redistribution. Because hydrogen and deuterium do not differ in electronic charge, such a difference cannot be attributed to the change in Coulombic interactions of the counterions, but to the change of the dipole moment associated with the hydrogen bridge, and possibly to their spatial delocalization as a result of NQEs.^103^

**Figure 5:**
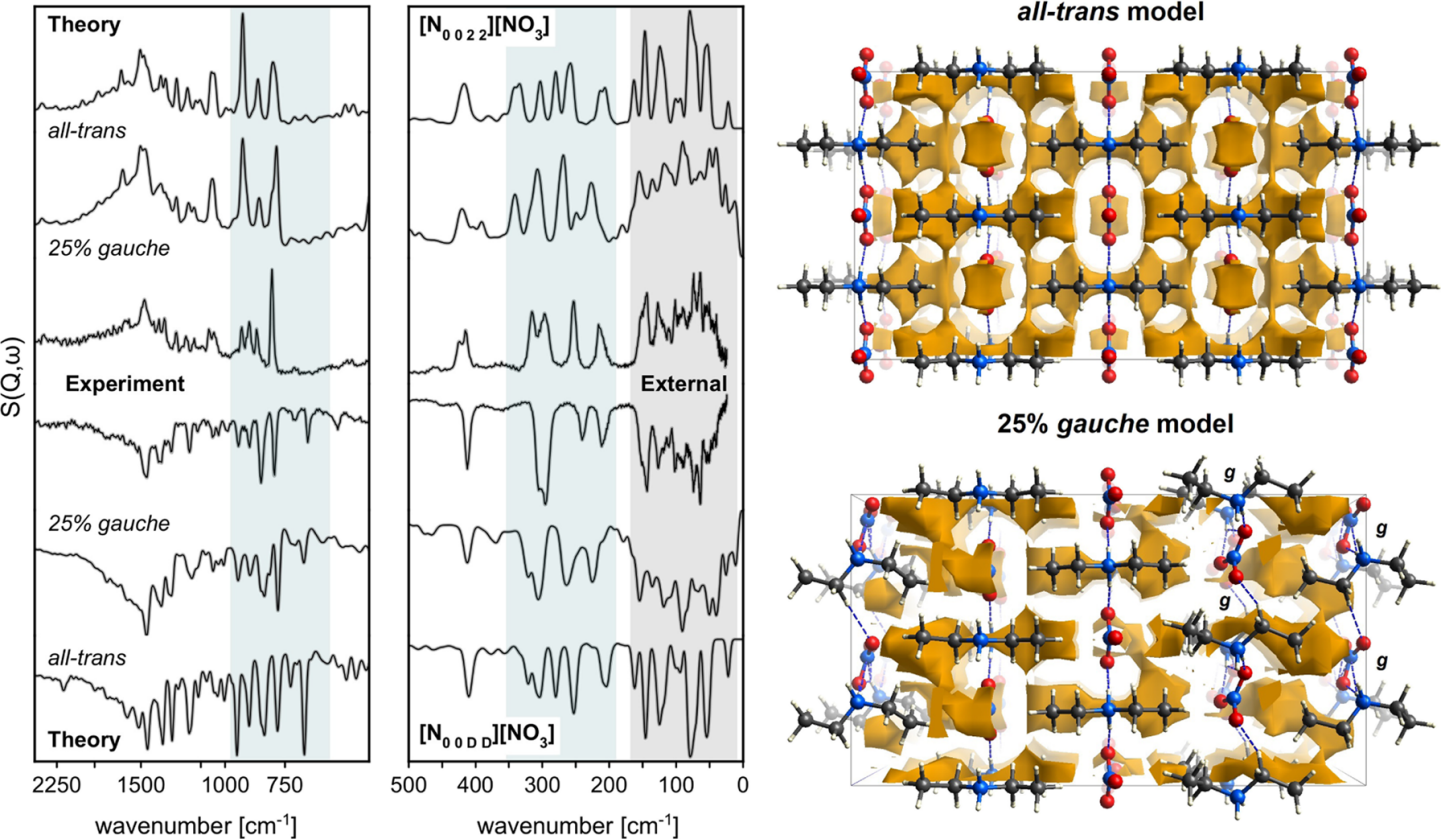
(Spectra on the left) Experimental and theoretical (harmonic lattice dynamics, PBE/1050 eV/hard-NCPPs) INS spectra of hydrogenous (↑) [N_0 0 2 2_][NO_3_] and perdeuterated (↓) [N_0 0 *D D*_][NO_3_] diethylammonium nitrate crystals in the intermediate- (1700–500 cm^–1^; the left panel) and low-energy regime (500–24 cm^–1^; the right panel), respectively. The theoretical spectra come from two different models, a perfect *Pmmn* crystal (all-trans, primitive-cell calculations) and partially disordered supercell model accounting for conformational changes in the powder specimen (25% gauche). The shaded area in cyan highlights the spectral range particularly sensible to conformational changes. The gray shaded area highlights the external modes. (Structural models on the right) Crystal voids are shown in brown over the 2 × 2 × 2 supercell of the crystallographic *Pmmn* model (all-trans), and compared to a model accounting for a 25% contribution from CH_2_CH_3_ chains in gauche conformation (*P*1 symmetry).

INS experiments entering the terahertz range of the spectrum (see Figure 5) give access to a region, where additional spectroscopic signatures of H-bonds between the anion and the cation are anticipated to appear.^104^ The calculated normal modes of [N_0 0 2 2_][NO_3_] have been visualized to identify the nature of the underlying vibrations (see links to the animation files in Table S1). Unlike IR/THz spectroscopy that probes the charge fluctuations around the electron-rich atoms in the hydrogen bridge, INS is primarily sensitive to the dynamics of hydrogen. Nonetheless, motions of the heavier atoms will still contribute indirectly to INS intensities. As clearly evidenced from the calculations (see Table S1), hydrogen-bridge stretching modes contribute below ca. 150 cm^–1^. These collective vibrations cover the upper part of the external-mode range highlighted in Figure 5 as a dark gray area.

Comparison of both experimental and theoretical spectra shows that the external-mode range is indistinguishable for both specimens, confirming that deuteration hardly affects the lattice modes. Furthermore, this result unequivocally confirms that the crystallographic structure of both systems studied in this work is the same. However, due to a prevalent amount of CH_2_CH_3_, the [ND_2_]^+^ motions provide only a minor contribution to the total spectral intensity in this spectral range. In a series of seminal works, Ludwig and co-workers have illustrated the ability of low-wavenumber optical spectroscopy (IR/THz-TDS) to detect the formation of H-bonding in several archetypal ILs, primarily those involving imidazolium ILs.^11,13−15,105−110^ It has been shown that the main IR/THz intensities typically occur at around 120 and 50 cm^–1^, being attributed to the stretching and deformation modes of the hydrogen bridges, respectively. By analyzing liquid samples, the authors have drawn a general observation stating that the stronger the H-bonding, the higher the mode energy and the corresponding IR activity. Interestingly, it has also been shown that the shifts to lower energies are mainly caused by a decrease in force constants, with only minor contributions from an increase in reduced mass (below 5 cm^–1^).^109,110^ Our calculations fully confirm the assignment presented by Ludwig, by scrutinizing the character of these low-energy vibrations to a much greater level of detail owing to a well-defined crystal environment and the low-temperature conditions of the INS experiments. The very high predictive ability of the adopted numerical methodology,^56,68,69,71^ with an uncertainty below 10 cm^–1^ (see Figure S9), places the characteristic ν[N···O] stretching of the hydrogen bridges at 118 cm^–1^, which is very close to the value discerned with INS at 10 K (110 cm^–1^). The small deviation from these two figures arises from several contributions, including: inherent errors of the semilocal XC functional; neglect of vdW corrections; artificial internal stresses from the fixed-cell methodology; as well as the harmonic approximation used in the phonon calculations.^111^ The energy of the ν[N···O] mode is expected to further decrease from the low-temperature limit with temperature,^109^ however, still allowing the classification of the strength of the H-bonding as moderate.^11,13−15,105−110^ For comparison, in the case of pyrrolidinium nitrate ([Pyrr][NO_3_]),^14^ or propylammonium nitrate ([PrAm][NO_3_]),^112^ Ludwig and coauthors have observed a ν[N···O] stretching mode as high as 200 cm^–1^, indicating a considerable increase of the H-bonding strength. This may suggest the formation the multicentered hydrogen bonds with a single hydrogen donor, a result in line with neutron diffraction experiments on an analogous pyrrolidinium acetate^113^ revealing that canonical single-contact H-bonds are not dominant motifs (contributions at the level of 25%).

The INS data also exhibit a number of well-defined features over the range 450–175 cm^–1^, which are primarily due to torsional deformations of the diethylammonium skeleton, and thus sensitive to isotopic substitution. A distinct doublet has been found in the [N_0 0 2 2_][NO_3_] sample at 424 and 415 cm^–1^, respectively. In the spectrum of the perdeuterated sample, only one single band with an enhanced intensity was found around 412 cm^–1^. These modes are assigned to an antisymmetric scissoring of the ethylammonium fragments, δ_asym_[C–C–N], resulting in a considerable motion of the hydrogen atoms along the direction transverse to the H-bond coordinate, combined with vibrational motions associated with the CH_3_ groups. The lower-frequency mode corresponds to the asymmetric motion of hydrogen in the CH_3_ groups in alternate molecular units along the crystallographic *b*-direction, where, for higher-frequency modes, these motions are all symmetric. From inspection of Table S1, it is clear that peaks at 412 and 408 cm^–1^ for the perdeuterated specimen are of the same symmetry and nature as the spectral features predicted at 419–414 cm^–1^ in the protonated samples. The peaks are red-shifted in the deuterated sample due to the higher mass of deuterium, become unresolved within instrumental resolution, and result in an increased band intensity. A similar effect is observed for the bands at 327 and 316 cm^–1^ (316 and 305 cm^–1^ in the case of [N_0 0 *D D*_][NO_3_]). The higher-energy feature 327(316) cm^–1^ is assigned to a γ[C–N–C] deformation, which affects the O···H distance and is quite intense from its mixing with large-amplitude τ[CH_3_] librations. The lower-frequency features at 316(305) cm^–1^ also involve τ[CH_3_] motions coupled to τ[C–N–C] chain twisting. The H-bond dynamics is also reflected in an isolated feature found at 253 and 240 cm^–1^ in [N_0 0 2 2_][NO_3_] and [N_*D D* 2 2_][NO_3_], respectively. This twisting cation deformation has also a strong contribution from bending modes transverse to the H-bond coordinate. In the deuterated crystal, this mode is red-shifted due to the increased mass of deuterium, and it is also suppressed in intensity. Furthermore, a well-defined feature found at 215 (H) and 210 cm^–1^ (D) reflects symmetric δ[C–N–C] bendings, which also modulate the O···H distance.

The above-discussed findings have also more important implications when compared to the ab initio predictions. By inspection of Figure 5, we note that the calculations performed in terms of the primitive cell with *Pmmn* symmetry are satisfying, yet some discrepancies remain when compared to the experimental data. The source of this mismatch can be in part ascribed to the limitations of the theoretical methodology. Nevertheless, our previous experience with similar systems^56,68,69,71^ along with the above-presented PXRD data suggests another scenario involving a certain degree of conformational freedom in the crystal structure. Such an effect would need to be taken into account to provide a complete structural picture of this material and an improved description of the INS data. To verify this hypothesis, we have examined several extended theoretical models capturing the conformational freedom in the crystallographic structure to varying degrees. To this end, we used a supercell of 16 diethylammonium cations and increased the number of gauche conformers in the model, as shown in Figure S12. The internal coordinates were fully relaxed, and the zone-center phonons were calculated with the finite-displacement method, using a displacement amplitude of 0.01 Å. The inspection of the resulting INS spectra shows that a model including 25% gauche conformers retaining the crystal-packing observed with SXRD provides a considerably improved description of the INS response. This result has been confirmed with the simulated PXRD patterns shown in Figure 2 along with the comparison with spectroscopic data presented in Figure 5. Accounting for conformational disorder provides an improved spectral match for both specimens of relevance to this work (see the cyan-shaded areas in the figure). This is particularly the case for the INS data in the range around 900 cm^–1^, involving large-amplitude ρ[NH_2_] modes. Also, some additional features such as the weak satellite bands around 350 and 450 cm^–1^ arise from torsional deformations of the gauche conformers. The conformational changes also affect the separation of the features observed in the range 350–200 cm^–1^ and provide an improved description of the external-mode range. However, these differences do not affect the general interpretation provided above, confirming that the long-range order is largely preserved in the solid state. These observations are also in line with the high directionality of cation–anion interactions, which is further evidence of H-bond formation. The ability of the adopted methodology to track subtle structural peculiarities also paves the way for further investigations aimed at interrogating the role of water in the disruption of the solid-state structures of highly hydroscopic ILs, or to address the question as to which extent a given conformation can persist in a highly disordered solid or amorphous environment, allowing further extrapolation of this knowledge onto the liquid state. INS, hence, emerges as a much-needed and complementary method to neutron diffraction techniques,^114,115^ to address these questions in PILs.

## 4 Conclusions

This work has reported the synthesis of a prototypical PIL system, diethylammonium nitrate, [N_0 0 2 2_][NO_3_], and its perdeuterated analogue, [N_0 0 *D D*_][NO_3_]. Both have been subjected to a detailed experimental scrutiny using XRD, calorimetry, and complementary IR, Raman, and high-resolution INS spectroscopy. The calorimetric study reveals the presence of two solid phases—a stable low-temperature phase up to 60 °C and a high-temperature phase—preceding the formation of the liquid at ca. 100 °C. The low-temperature phase has been characterized by both SXRD and PXRD, and is characterized by a high-symmetry structure within the *Pmmn* orthorhombic space group. This structure can accommodate the formation of H-bonding motifs between the counterions. A semiquantitative analysis of intermolecular interactions highlights the importance of dispersion forces stabilizing a staggered conformation of the cation in this densely packed phase. The low-temperature phase has been subjected to extensive vibrational spectroscopy analysis, heavily supported by theoretical calculations.

The first-principles calculations have been performed within the framework of semilocal plane-wave DFT (PBE), providing a complete normal-mode vibrational analysis. The IR, Raman, and INS spectra calculated within the harmonic approximation compare very well with the experimental data for most of the assigned spectral features but the ones involving the [NH_2_] motions, revealing a pronounced anharmonicity. Furthermore, non-trivial effects associated with deuteration can be explained using these calculations. Chemically speaking, we have identified vibrational modes associated with hydrogen-bonding interactions, manifesting themselves mainly at intermediate energies. Mechanical anharmonicity has been assessed by means of ab initio MD simulations using effective temperatures. These calculations provide an improved description of the vibrational energies, significantly reducing the deficiencies associated with the harmonic approximation. The MD simulations also reveal a high propensity of the nitrate ions to disorder, accompanied by the conformational changes within the alkyl parts of the counterion, thus providing new insights into the local structure of the high-temperature, conformationally disordered phase. Further theoretical calculations rule out possible scenarios involving proton migration in the low-temperature phase or the presence of nitric acid^98^ and ethylamine, in line with experimental observations.

An exhaustive analysis of the INS data has allowed us to identify the signatures of hydrogen-bonding well within the terahertz-regime. We also find that external modes are equivalent for both specimens, indicating that isotopic effects have no noticeable influence on the crystal structure. The INS data also provide important clues on the orientational disorder ubiquitous in ILs, yet at the same time these do not seem to affect the nature of the hydrogen-bonding interactions identified in this work. A joint analysis of the low- and high-energy spectral regimes performed by means of optical and neutron spectroscopies allows us to classify the observed H-bonding motifs as of moderate strength.

In spite of the intrinsic limitations of the PBE functional, the adopted numerical methodology has proven to be entirely adequate for a robust physicochemical interpretation of the vibrational spectra in a well-defined crystal environment. In hindsight, the wealth of experimental data collected in this work could further serve as a high-quality benchmark for much-needed developments of accurate classical force-fields for PILs,^116−119^ thereby paving the way for temporally and spatially resolved MD simulations transcending a well-defined crystal environment or the quality of semilocal DFT functionals.

## Supporting Information Available

Additional structural information; results of DSC and POM measurements; additional computational results; and a detailed spectral analysis including normal-mode animations associated with each vibration of interest (PDF)
SXRD structure of the hydrogenous specimen (CIF)
Outputs from phonon calculations for the visualization of eigenvectors (ZIP)

## Supplementary Material

jp1c05137_si_001.pdf

jp1c05137_si_002.cif
