# Supplementary material for: Spectroscopic Signatures of Hydrogen-Bonding Motifs in Protonic Ionic Liquid Systems: Insights from Diethylammonium Nitrate in the Solid State
Source: J Phys Chem C Nanomater Interfaces. 2021 Oct 27;125(44):24463–76. doi: 10.1021/acs.jpcc.1c05137 (PMC8592064; doi:10.1021/acs.jpcc.1c05137)
Supplement: Supplementary file 1 — jp1c05137_si_001.pdf [file jp1c05137_si_001.pdf]

Supplementary Information for Publication: Spectroscopic Signatures of Hydrogen-bonding Motifs in Protonic Ionic-Liquid Systems: Insights from Diethylammonium Nitrate in the Solid State

Isabel Vázquez-Fernández,[a] Kacper Drużbicki,*[,b,c] Felix Fernandez-Alonso,[b,d,e,f] Sanghamitra Mukhopadhyay,[g,h] Peter Nockemann,[a] Stewart F. Parker,[g] Svemir Rudić,[g] Simona-Maria Stana,[a] John Tomkinson,[g] Darius J. Yeadon,[a] Kenneth R. Seddon,*[,a] and Natalia V. Plechkova*[,a]

[a]The QUILL Research Centre, School of Chemistry and Chemical Engineering, The Queen's University of Belfast, Belfast, BT9 5AG, Northern Ireland, United Kingdom.
E-mail: kacper.druzbicki@ehu.eus; n.plechkova@qub.ac.uk
[b]Materials Physics Center, CSIC-UPV/EHU, Paseo Manuel Lardizábal 5, 20018 Donostia-San Sebastian, Spain;
[c]Centre of Molecular and Macromolecular Studies, Polish Academy of Sciences, Sienkiewicza 112, 90-363 Lodz, Poland;
[d]Donostia International Physics Center (DIPC), Paseo Manuel de Lardizabal 4, 20018 Donostia, San Sebastian, Spain;
[e]Department of Physics and Astronomy, University College London, Gower Street, London WC1E 6BT, United Kingdom;
[f]IKERBASQUE, Basque Foundation for Science, Plaza Euskadi 5, 48009 Bilbao, Spain;
[g]ISIS Facility, Rutherford Appleton Laboratory, Chilton, Didcot, OX11 0QX, United Kingdom;
[h]Department of Materials, Imperial College London, Exhibition Road, London, SW72AZ, United Kingdom

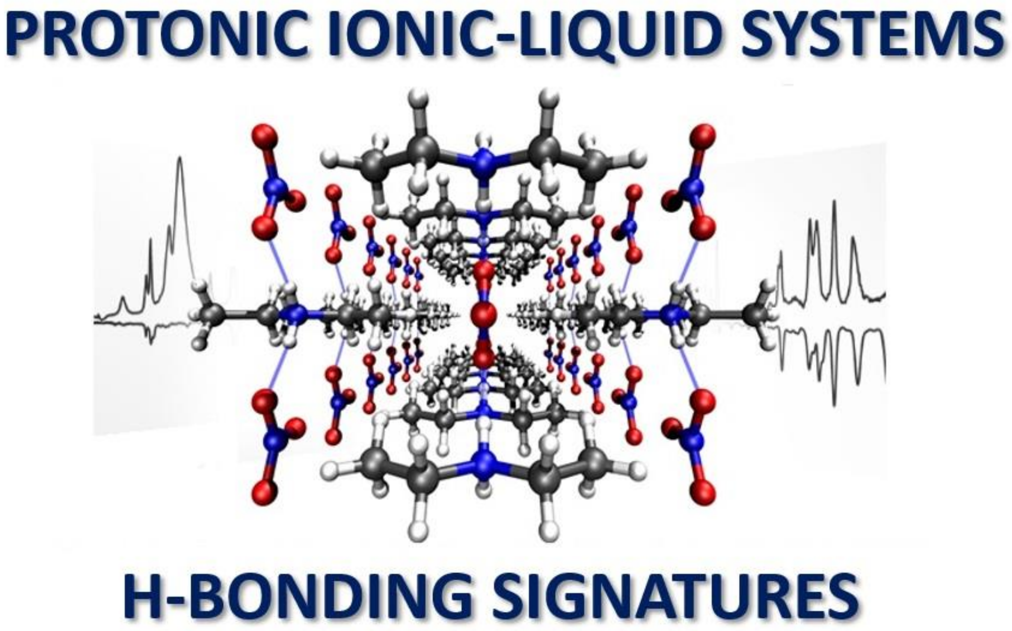

S1. Supplementary Structural Information

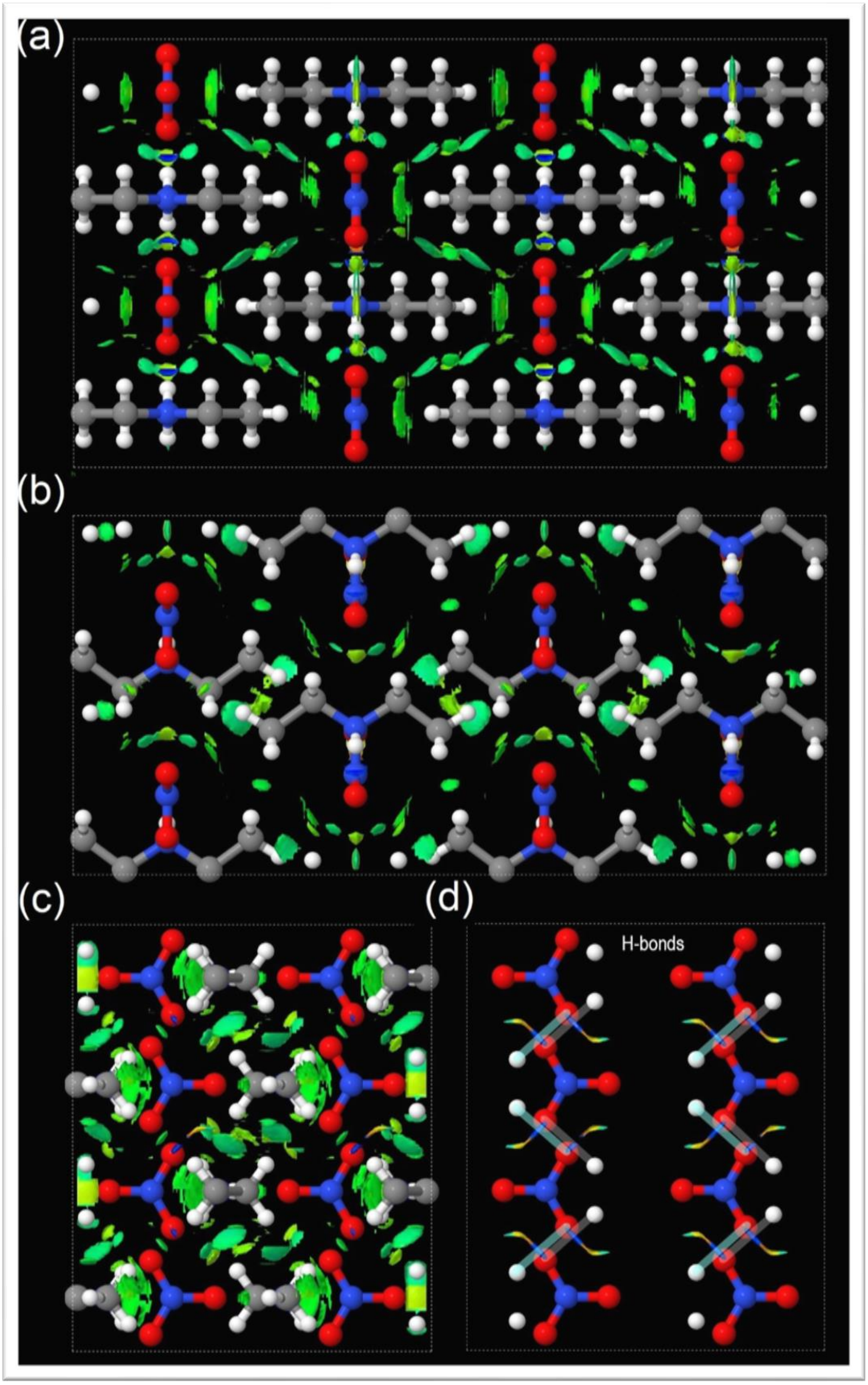

Figure S1 Graphical summary of the Non-Covalent Interaction (NCI) analysis of the orthorhombic *Pmmn* structure of diethylammonium nitrate, [N_{0022}][NO_3]. (a - c) 2-D projections of the pro-molecular NCI index within a 2x2x2 supercell. Gradient isosurfaces (s = 0.5 a.u.) of sign(λ_2)ρ over the range -0.03 to 0.03 a.u are colored using a BGR scale. With this choice, attractive and repulsive interactions correspond to different colors: blue for attractive, such as H-bonds; green for weak attractive interactions such as van-der-Waals (vdW); and red for steric repulsion. For clarity, the identified H-bond interactions are redrawn in panel (d). These are represented by transparent lines, masking all the atoms in the [N_{0022}] groups except the hydrogens.

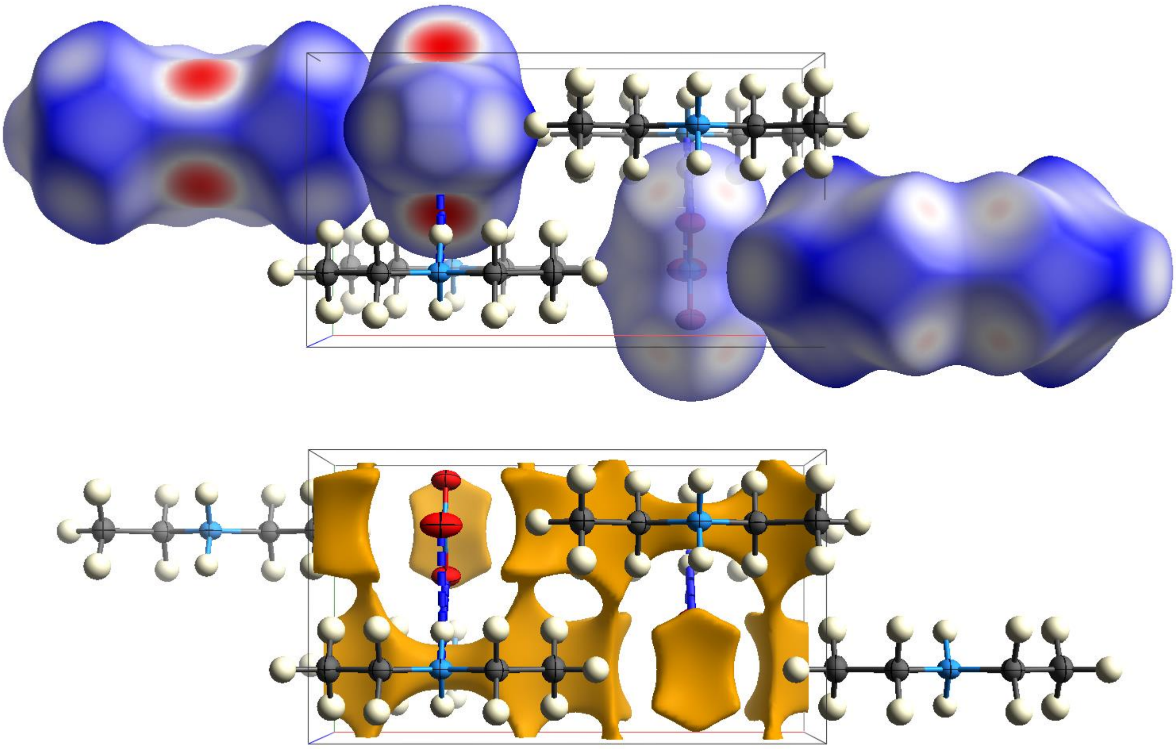

Figure S2 Hirshfeld surface (upper panel) and crystal voids (bottom panel) within the orthorhombic *Pmmn* unit cell of diethylammonium nitrate, $[N_{0022}][NO_3]$. The surfaces in the upper panel correspond to the contact distance (d_{norm}) to the nearest external atom. The intense red spots indicate the presence of H-bonding interactions. The blue regions correspond to longer contacts with positive d_{norm}, and white regions correspond to contacts exactly equal to the van-der-Waals (vdW) radii of the relevant atoms. Note the thermal ellipsoids on the oxygen atoms in the bottom panel suggesting pronounced out-of-plane displacements of the $[NO_3^-]$ anions.

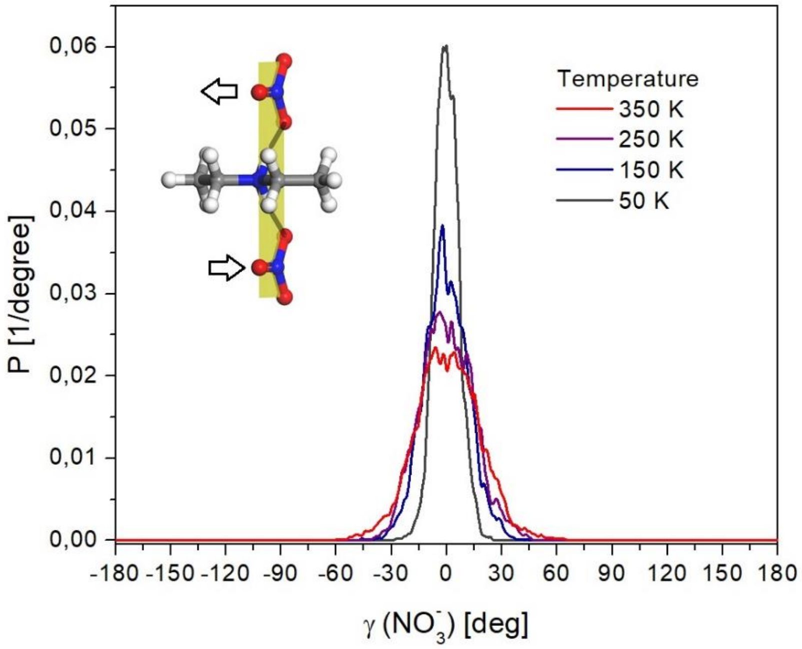

Figure S3 Distribution of γ(N-O-N-O) dihedral angles as a function of temperature, used to assess the relative orientation of the counterions in the crystal structure of diethylammonium nitrate, $[N_{0022}][NO_3]$. These results have been obtained from 25-ps *ab initio* MD simulations in the microcanonical ensemble. The molecular structure in the figure corresponds to a dihedral angle of zero, where the $[NO_3^-]$ anions lay in the plane perpendicular to the mirror-symmetry plane of the diethylammonium cations (point group C_{2v}). At the higher temperatures, these computational results show a marked propensity for out-of-plane motions of the $[NO_3^-]$ anions.

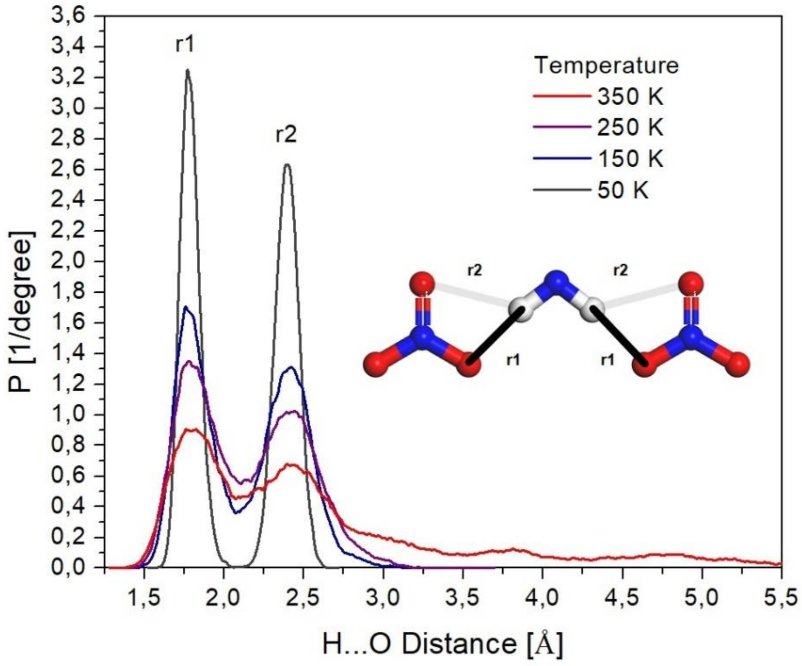

Figure S4 Temperature evolution of the distribution of O…H distances in the crystal structure of diethylammonium nitrate, [N_{0022}][NO_3]. These results have been obtained from 25-ps *ab initio* MD simulations in the microcanonical ensemble. The cartoon shows the definition of the two primary O…H distances. The presence of non-zero contributions above 3.0 Å at 350 K signals an increased propensity for in-plane motions of the [NO_3^-] anions at this temperature.

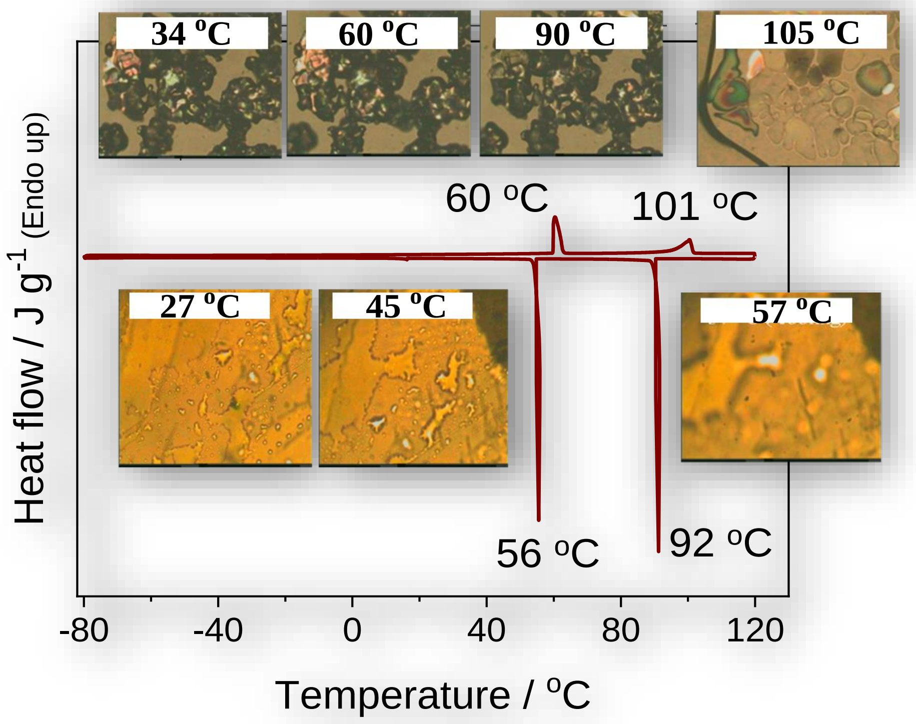

Figure S5 DSC scan of a hydrogenous [N_{0022}][NO_3] specimen at a heating and cooling rate of 5 °C/min under N_2, along with the corresponding polarized-optical-microscopy images.

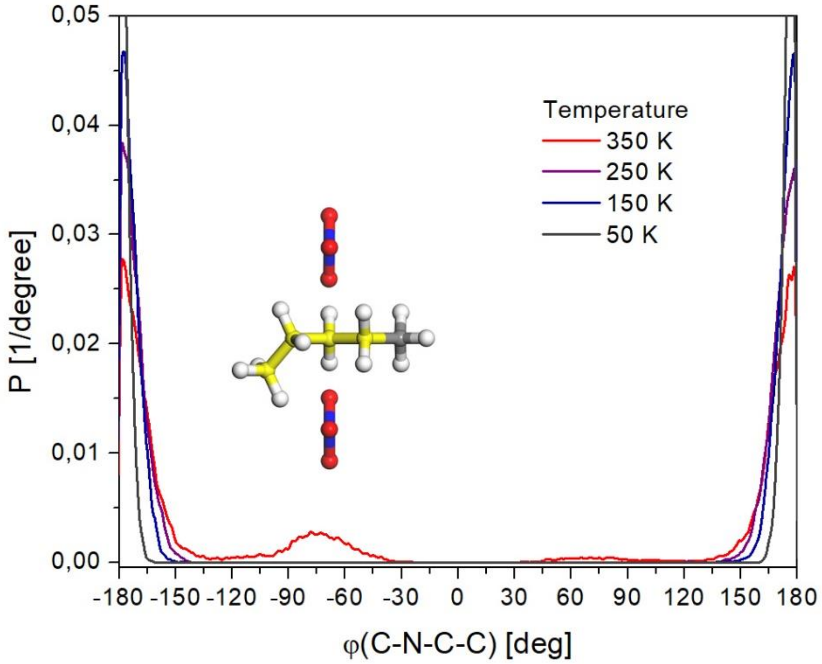

Figure S6 Distribution of φ(C-N-C-C) dihedral angles as a function of temperature, defining the conformation of the diethylammonium cations in the crystal structure of diethylammonium nitrate, $[N_{0\,0\,2\,2}][NO_3]$. These results have been obtained from 25-ps *ab initio* MD simulations in the microcanonical ensemble. At the highest temperature, there is a partial conformational change from *all-trans* to *gauche*. The resulting *gauche* conformer are shown in the cartoon.

S2. Spectral Analysis

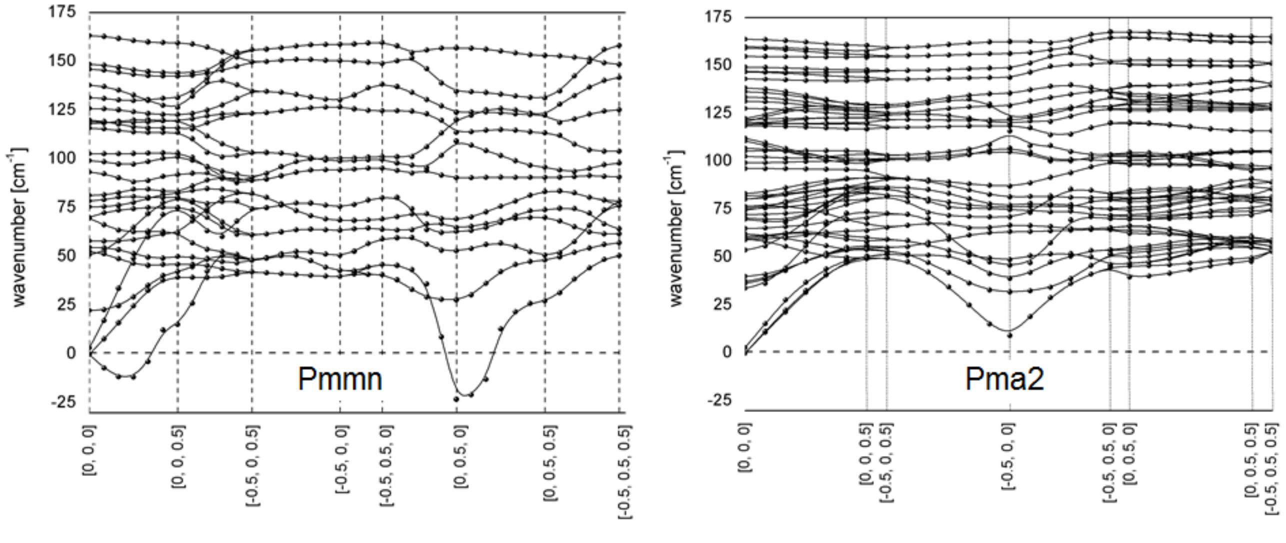

Figure S7 Left: Phonon dispersion curves (fixed-cell PBE/1050eV/ hard-NCPPs) calculated for the *Pmmn* model of hydrogenous $[N_{0\,0\,2\,2}][NO_3]$. Right: corresponding data for the *Pma2* structure, obtained by following the mechanical instability at [0 0.5 0.5] shown in the left panel. The resulting *Pma2* structure is stable. This mechanical instability occurs beyond the Γ-point and might be caused by internal artificial stresses in the fixed-cell calculations. The Γ-point phonon frequencies of relevance to this work (above 175 cm^{-1}) for both models are virtually-identical, as shown in Figure S8.

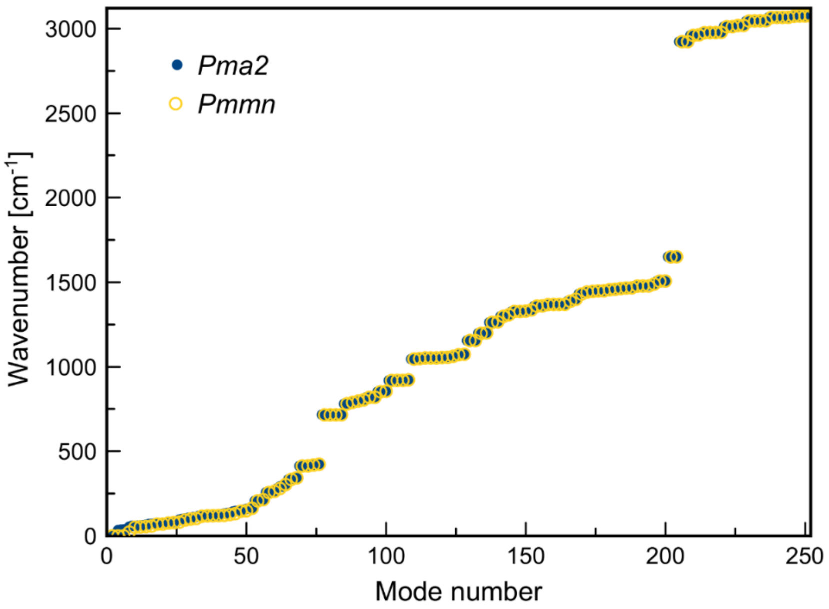

Figure S8 Comparison of the mode frequencies calculated with the same computational methodology for the *Pmmn* and the supercell *Pma2* models. See also Fig. S7.

Normal Mode Analysis

For the minimal-size model of the structure, there are 42 ions in the unit cell, which give rise to 123 non-zero frequencies *at the Γ-point.* The space group of the unit cell is *Pmmn* (No. 59), its point group is D_{2h}[13] and *Z* = 2, and the associated symmetries are:

$\Gamma_{red} = 19A_g + 11B_{1g} + 16B_{2g} + 17B_{3g} + 11A_u + 19B_{1u} + 17B_{2u} + 16B_{3u}$

$\Gamma_{acoustic} = B_{1u} + B_{2u} + B_{3u}$

$\Gamma_{silent} = 11A_u$

The results of fixed-cell PBE PW-DFT calculations are analyzed in detail below. Web links to interactive animations for a hydrogeneous [$N_{0\,0\,2\,2}$][NO_3] can be found in Table S1 as hyperlinks. We also provide the original phonon output files for both and [$N_{0\,0\,2\,2}$][NO_3], which are easily readable by an open-source software Jmol.

To visualize the modes, please:

a.) Download Jmol (http://jmol.sourceforge.net/download/)
b.) Go to the download folder and execute 'Jmol.jar'
c.) Open console (File / Console) and load the *.phonon file, for example:
zap; load SYNC "C: /H-DEAN-HLD.phonon" supercell {1 1 1}
zap; load SYNC "C: /D-DEAN-HLD.phonon" supercell {1 1 1}
d.) Animate phonons (Tools / Vibrate)

Table S1 *Γ*-point vibrational frequencies (cm^{-1}) in diethylammonium nitrate from fixed-cell calculations (CASTEP/PBE/NCPP/1050eV). Mode animations are given for a {1 2 2} supercell of a hydrogeneous [N_{0022}][NO_3]. The reported accuracy of mode energies reflects that of the calculations per se and it is only used to differentiate between close-to-degenerate normal modes.

	Hydrogeneous							Perdeuterated					
No.	PBE	INS	IR	Raman	Anim.	Description	Symm.	Symm.	PBE	INS	IR	Raman	Description
	CASTEP	EXPERIMENTAL							CASTEP	EXPERIMENTAL			
1	0.00				A	acoustic			0.00				acoustic
2	0.00				A	acoustic			0.00				acoustic
3	0.00				A	acoustic			0.00				acoustic
4	22.47	39			A					39			shearing mode (IP NO_3^- translation)
5	50.93				A				50.54				shearing mode (IP NO_3^- translation)
6	52.68	54			A	lib. NO_3^- (OP twist)				53			lib. NO_3^- (OP twist)
7	54.78				A	lib. NO_3^- (OP twist)			54.78				lib. NO_3^- (OP twist)
8	58.05				A	lib. Cation			57.89				lib. Cation
9	69.66				A	lib. NO_3^- / lib. Cation			69.66				lib. NO_3^- / lib. Cation
10	70.22	63			A	lib. NO_3^- / lib. Cation				65			lib. NO_3^- / lib. Cation
11	75.50				A	lib. NO_3^- / τ Cation (CH_2CH_3)			75.48				lib. NO_3^- / τ Cation (CH_2CH_3)
12	78.54				A				77.89				squashing mode (OP NO_3^- translation)
13	81.43	74			A	τ Cation (CH_2CH_3)				74			τ Cation (CH_2CH_3)
14	93.32	90			A					90			lib. NO_3^- (IP twist) / Cation wing wave
15	99.25				A				98.93				NO_3^- / Cation swinging mode (OP)
16	102.81	102			A					101			NO_3^- / Cation swinging mode (OP)
17	115.87				A	lib. NO_3^- (OP twist) / τ Cation			115.68				lib. NO_3^- (OP twist) / τ Cation
18	118.11	110			A	ν_{sym} (N···O) H-Bond				111			ν_{sym} (N···O) H-Bond
19	119.16				A	lib. NO_3^- (OP twist) / τ Cation			119.13				lib. NO_3^- (OP twist) / τ Cation
20	119.99				A				119.84				lib. NO_3^- (IP twist) / ν_{asym} (N···O) H-Bond
21	121.21				A	ν_{sym} (N···O) H-Bond			120.94				ν_{sym} (N···O) H-Bond
22	125.92	128			A	τ Cation (CH_2CH_3)				126			τ Cation (CH_2CH_3)
23	131.53				A				130.61				lib. NO_3^- (IP twist) / ν_{asym} (N···O) H-Bond
24	146.09	145			A	τ Cation (C-N-C twist)				143			τ Cation (C-N-C twist)
25	148.64				A				147.53				ν_{asym} (N···O) H-Bond / lib. NO_3^- (IP twist)
26	163.28	153			A	τ Cation (C-N-C twist)				152			τ Cation (C-N-C twist)
27	206.89			205	A	δ_{sym} Cation (C-C-N)			203.98			207	δ_{sym} Cation (C-C-N)
28	214.09	215			A	δ_{sym} Cation (C-C-N)				210			δ_{sym} Cation (C-C-N)
29	256.44	253			A	τ(CH_2CH_3) + τ(CH_3)				240			τ(CH_2CH_3) + τ(CH_3)
30	263.07				A	τ(CH_2CH_3) + τ(CH_3)			256.40				τ(CH_2CH_3) + τ(CH_3)
31	280.25	296			A	τ(CH_3) + τ Cation (C-N-C twist)				296			τ(CH_3) + τ Cation (C-N-C twist)
32	303.34	316			A	τ(CH_3) + τ Cation (C-N-C twist)				305			τ(CH_3) + τ Cation (C-N-C twist)

33	333.77				A				310.10				γ(C-N-C) H-Bond sensitive + $\tau(CH_3)$
34	343.22	327			A								γ(C-N-C) H-Bond sensitive + $\tau(CH_3)$
35	413.19				A				407.69				δ_{asym} Cation (C-C-N)
36	413.68	415		425/414	A				411.57				δ Cation - accordion mode δ (C-N-C)
37	418.62	424			A	δ_{asym} Cation (C-C-N)				412		424/416	δ Cation - accordion mode δ (C-N-C)
38	423.46				A	δ_{asym} Cation (C-C-N)			412.41				δ_{asym} Cation (C-C-N)
39	715.01				A	δ (NO_3^-) [v_6 mode]			683.24				$\rho(NH_2)$ + $\rho(CH_2)$ / $\delta(NO_3^-)$ [v_6 mode]
40	715.27				A	δ_{sym} (NO_3^-) [v_3 mode] / $\rho(NH_2)$				672 (d)			$\rho(NH_2)$ + $\rho(CH_2)$ / $\delta(NO_3^-)$ [v_6 mode]
41	715.29			719	A	δ_{sym} (NO_3^-) [v_3 mode] / $\rho(NH_2)$			714.83			715	δ_{sym} (NO_3^-) [v_3 mode]
42	715.75		715		A	δ_{sym} (NO_3^-) [v_3 mode]			715.61		710		δ_{sym} (NO_3^-) [v_3 mode]
43	780.76				A	$\rho(CH_3)$ + $\rho(CH_2)$ + $\tau(NH_2)$			729.18		717		$\rho(NH_2)$ + $\rho(CH_2)$ / $\delta(NO_3^-)$ [v_5 mode]
44	789.20				A	$\rho(CH_3)$ + $\rho(CH_2)$ + $\tau(NH_2)$			729.19			719	$\rho(NH_2)$ + $\rho(CH_2)$ / $\delta(NO_3^-)$ [v_5 mode]
45	796.80		791		A	$\rho(CH_3)$ + $\rho(CH_2)$ + $\rho(NH_2)$			773.79				$\rho(CH_3)$ + $\rho(CH_2)$ + $\tau(NH_2)$
46	804.46				A	$\rho(CH_3)$ + $\rho(CH_2)$ + $\rho(NH_2)$			781.33				$\rho(CH_3)$ + $\rho(CH_2)$ + $\tau(NH_2)$
47	819.27		826		A	$\gamma(NO_3^-)$ [v_6 mode]			819.18		827		$\gamma(NO_3^-)$ [v_6 mode]
48	819.73				A	$\gamma(NO_3^-)$ [v_6 mode]			819.50				$\gamma(NO_3^-)$ [v_6 mode]
49	852.46		856		A	ν_{sym} (C-N-C) + $\rho(CH_3)$			822.30		834		$\rho(CH_3)$ + $\rho(CH_2)$ + $\rho(NH_2)$
50	855.15			867/860	A	ν_{sym} (C-N-C) + $\rho(CH_3)$			830.76				$\rho(CH_3)$ + $\rho(CH_2)$ + $\rho(NH_2)$
51	918.58				A	$\rho(NH_2)$ + $\tau(CH_2)$ + $\rho(NH_2)$			841.98		827		ν_{sym} (C-N-C) + $\rho(CH_3)$
52	919.24	924 (d)			A	$\rho(NH_2)$ + $\tau(CH_2)$ + $\rho(NH_2)$			844.20			868/856	ν_{sym} (C-N-C) + $\rho(CH_3)$
53	919.28				A	ν_{asym} (C-N-C) + $\rho(CH_3)$			888.19				ν_{asym} (C-N-C) + $\rho(CH_3)$
54	922.72			925	A	ν_{asym} (C-N-C) + $\rho(CH_3)$			891.21				ν_{asym} (C-N-C) + $\rho(CH_3)$
55	1045.57				A	ν_{sym} (C-N-C)			944.34				$\tau(NH_2)$ + $\tau(CH_2)$ + $\rho(CH_3)$
56	1048.43				A	ν_{sym} (C-N-C)				939		923	$\tau(NH_2)$ + $\tau(CH_2)$ + $\rho(CH_3)$
57	1052.03				A	ν_{sym} (NO_3^-) [v_2 mode]					995 (c)		$\gamma(NH_2)$ / γ (NH···O) H-Bond + ν_{asym} (C-C)
58	1052.15			1043	A	ν_{sym} (NO_3^-) [v_2 mode]			1007.93			998 (c)	$\gamma(NH_2)$ / γ (NH···O) H-Bond + ν_{asym} (C-C)
59	1052.18	1055			A	$\rho(CH_3)$ / ν_{asym} (C-N-C)				1032			ν_{sym} (C-C)
60	1054.18		1057/1049		A	$\rho(CH_3)$ / ν_{asym} (C-N-C)			1038.25				ν_{sym} (C-C)
61	1055.22				A	$\rho(CH_3)$ / ν_{asym} (C-N-C)			1051.46				ν_{sym} (NO_3^-) [v_2 mode]
62	1061.64				A	$\rho(CH_3)$ / ν_{asym} (C-N-C)			1051.82			1045	ν_{sym} (NO_3^-) [v_2 mode]
63	1071.25				A	$\tau(NH_2)$ + $\rho(CH_2)$ + $\rho(CH_3)$			1052.77		1055		$\rho(CH_3)$ / ν_{asym} (C-N-C)
64	1073.76	1080			A	$\tau(NH_2)$ + $\rho(CH_2)$ + $\rho(CH_3)$				1062			$\rho(CH_3)$ / ν_{asym} (C-N-C)
65	1154.99		1158		A	$\rho(CH_3)$ + δ_{sym} Cation (C-C-N)			1136.13		1137		$\delta(NH_2)$ + $\rho(CH_3)$ + δ_{sym} Cation (C-C-N)
66	1155.22	1160		1164	A	$\rho(CH_3)$ + δ_{sym} Cation (C-C-N)				1142			$\delta(NH_2)$ + $\rho(CH_3)$ + δ_{sym} Cation (C-C-N)
67	1197.13	1208	1208		A	$\rho(NH_2)$ + $\rho(CH_2)$ + $\rho(CH_3)$			1172.63		1156		$\rho(CH_3)$ + $\rho(CH_2)$ + $\rho(NH_2)$
68	1200.10			1208	A	$\rho(NH_2)$ + $\rho(CH_2)$ + $\rho(CH_3)$				1186		1162	$\rho(CH_3)$ + $\rho(CH_2)$ + $\rho(NH_2)$
69	1262.61	1265			A	$\tau(NH_2)$ + $\tau(CH_2)$ + $\rho(CH_3)$			1176.16				$\tau(NH_2)$ + $\rho(CH_2)$ + $\rho(CH_3)$
70	1265.52			1270	A	$\tau(NH_2)$ + $\tau(CH_2)$ + $\rho(CH_3)$			1178.66				$\tau(NH_2)$ + $\rho(CH_2)$ + $\rho(CH_3)$

71	1298.12	1313	1308		A	$\rho(NH_2) + \tau(CH_2)$	[B3u]	[B2u]	1190.44				$\omega(NH_2) + \omega(CH_2) + \rho(CH_3)$
72	1307.89				A	$\rho(NH_2) + \tau(CH_2)$	[B2g]	[B3g]	1191.57				$\omega(NH_2) + \omega(CH_2) + \rho(CH_3)$
73	1327.16				A	$\delta(NH_2)$ / $\delta(CH_2)$ / $\rho(CH_3)$ / $\nu_{asym}(NO_3^-)$ [v_4 mode]	[Ag]	[Ag]	1194.40			1185 (b)	$\delta(NH_2)$ / $\delta(NH \cdots O)$ H-Bond
74	1327.17				A	$\delta(NH_2)$ / $\delta(CH_2)$ / $\rho(CH_3)$ / $\nu_{asym}(NO_3^-)$ [v_4 mode]	[B1u]	[B1u]	1194.85		1180 (b)		$\delta(NH_2)$ / $\delta(NH \cdots O)$ H-Bond
75	1329.42	1339			A	$\omega(NH_2) + \omega(CH_2) + \rho(CH_3)$	[B3g]	[B3u]	1287.37	1296			$\tau(CH_2)$
76	1335.42				A	$\omega(NH_2) + \omega(CH_2) + \rho(CH_3)$	[B2u]	[Au]	1287.54		1304		$\tau(CH_2)$
77	1358.60				A	$\rho(NH_2) + \tau(CH_2)$ / $\nu_{asym}(NO_3^-)$ [v_4 mode]	[B2g]	[B1g]	1293.51				$\tau(CH_2)$
78	1359.91	1376	1360		A	$\rho(NH_2) + \tau(CH_2)$ / $\nu_{asym}(NO_3^-)$ [v_4 mode]	[B3u]	[B2g]	1297.50			1302	$\tau(CH_2)$
79	1367.87				A	$\delta_{sym}(CH_3) + \omega(CH_2)$ / $\nu_{asym}(NO_3^-)$ [v_1 mode]	[B1u]	[B3g]	1345.52	1358			$\omega(CH_2)$
80	1368.60				A	$\delta_{sym}(CH_3) + \omega(CH_2)$	[B3g]	[B2g]	1347.39				$\tau(CH_2)$
81	1368.76				A	$\delta_{sym}(CH_3) + \omega(CH_2)$	[B2u]	[B3u]	1348.89		1346		$\tau(CH_2)$ / $\nu_{asym}(NO_3^-)$ [v_4 mode]
82	1368.84				A	$\delta_{sym}(CH_3) + \omega(CH_2)$ / $\nu_{asym}(NO_3^-)$ [v_1 mode]	[Ag]	[Ag]	1351.23				$\delta_{sym}(CH_3) + \omega(CH_2)$ / $\nu_{asym}(NO_3^-)$ [v_1 mode]
83	1387.43			1377	A	$\omega(CH_2) + \delta_{sym}(CH_3)$	[Ag]	[B2u]	1353.00				$\omega(CH_2)$
84	1399.22	1411	1405		A	$\omega(CH_2) + \delta_{sym}(CH_3)$ / $\nu_{asym}(NO_3^-)$ [v_1 mode]	[B1u]	[B1u]	1354.35				$\nu_{asym}(NO_3^-)$ [v_1 mode] / $\delta_{sym}(CH_3)$
85	1430.07		1441		A	$\tau(NH_2) + \tau(CH_2) + \delta(CH_3)$	[Au]	[B2u]	1368.77				$\delta_{sym}(CH_3) + \omega(CH_2)$
86	1442.33			1442	A	$\tau(NH_2) + \tau(CH_2) + \delta(CH_3)$	[B1g]	[B3g]	1368.86				$\delta_{sym}(CH_3) + \omega(CH_2)$
87	1446.07				A	$\delta_{asym}(CH_3) + \delta(CH_2)$	[B3g]	[B1u]	1369.20	1377			$\delta_{sym}(CH_3) + \omega(CH_2)$ / $\nu_{asym}(NO_3^-)$ [v_1 mode]
88	1448.88				A	$\delta_{asym}(CH_3)$	[B2g]	[Ag]	1373.84				$\delta_{sym}(CH_3) + \omega(CH_2)$ / $\nu_{asym}(NO_3^-)$ [v_1 mode]
89	1449.67			1456	A	$\delta_{asym}(CH_3) + \omega(CH_2)$	[Ag]	[Ag]	1385.12				$\delta_{sym}(CH_3) + \omega(CH_2)$
90	1455.39		1459		A	$\delta_{asym}(CH_3)$	[B3u]	[B1u]	1397.01	1412	1406		$\delta_{sym}(CH_3) + \omega(CH_2)$
91	1457.99				A	$\delta(CH_2)$	[B2u]	[B3g]	1447.02			1443	$\delta_{asym}(CH_3) + \omega(CH_2)$
92	1462.22			1467	A	$\delta(CH_2)$	[B3g]	[B2g]	1448.87				$\delta_{asym}(CH_3)$
93	1465.93				A	$\delta_{asym}(CH_3) + \delta(CH_2)$	[B2u]	[B1g]	1449.26				$\delta_{asym}(CH_3)$
94	1466.93		1475		A	$\delta_{asym}(CH_3)$	[B1u]	[Ag]	1450.59			1457	$\delta_{asym}(CH_3) + \omega(CH_2)$
95	1476.96				A	$\delta(CH_2)$	[Ag]	[Au]	1453.18				$\delta_{asym}(CH_3)$
96	1477.29	1474			A	$\tau(NH_2)$	[B1g]	[B3u]	1454.98		1460		$\delta_{asym}(CH_3)$
97	1477.52			1483	A	$\delta(CH_2)$	[B1u]	[B2u]	1459.00				$\delta(CH_2)$
98	1489.12				A	$\tau(NH_2)$	[Au]	[B3g]	1462.51				$\delta(CH_2)$
99	1506.29			1459 (c)	A	$\gamma(NH_2)$ / $\gamma(NH \cdots O)$ H-Bond + ν(C-N)	[B3g]	[B2u]	1466.87				$\delta_{asym}(CH_3) + \delta(CH_2)$
100	1506.92		1483		A	$\gamma(NH_2)$ / $\gamma(NH \cdots O)$ H-Bond + ν(C-N)	[B2u]	[B1u]	1468.98				$\delta_{asym}(CH_3)$
101	1649.83			1613 (b)	A	$\delta(NH_2)$ / $\delta(NH \cdots O)$ H-Bond	[Ag]	[Ag]	1476.98			1486	$\delta(CH_2)$
102	1650.64		1613 (b)		A	$\delta(NH_2)$ / $\delta(NH \cdots O)$ H-Bond	[B1u]	[B1u]	1478.11	1483			$\delta(CH_2)$
103	2922.88				A	$\nu_{asym}(NH_2)$	[B2g]	[Ag]	2162.27				$\nu_{sym}(NH_2)$
104	2922.89		2853 (a)		A	$\nu_{asym}(NH_2)$	[B3u]	[B1u]	2164.30		2155 (a)		$\nu_{sym}(NH_2)$
105	2959.37				A	$\nu_{sym}(NH_2)$	[Ag]	[B3u]	2184.49		2252 (a)		$\nu_{asym}(NH_2)$
106	2962.69		3049 (a)		A	$\nu_{sym}(NH_2)$	[B1u]	[B2g]	2184.58				$\nu_{asym}(NH_2)$
107	2975.56				A	$\nu_{sym}(CH_3)$	[B3g]	[B3g]	2975.55				$\nu_{sym}(CH_3)$
108	2976.19			2896	A	$\nu_{sym}(CH_3)$	[Ag]	[Ag]	2975.84			2894	$\nu_{sym}(CH_3)$

109	2976.31				A	ν_{sym} (CH_3)	[B2u]	[B2u]	2976.29				ν_{sym} (CH_3)
110	2977.44				A	ν_{sym} (CH_3)	[B1u]	[B1u]	2976.70				ν_{sym} (CH_3)
111	3011.44				A	ν_{sym} (CH_2)	[B2u]	[B2u]	3011.42				ν_{sym} (CH_2)
112	3011.68				A	ν_{sym} (CH_2)	[B3g]	[B3g]	3011.66				ν_{sym} (CH_2)
113	3018.16		2970		A	ν_{sym} (CH_2)	[B1u]	[B1u]	3015.12		2970		ν_{sym} (CH_2)
114	3018.67			2953	A	ν_{sym} (CH_2)	[Ag]	[Ag]	3015.35			2951	ν_{sym} (CH_2)
115	3044.46			2998	A	ν_{asym} (CH_3) + ν_{asym} (CH_2)	[B1g]	[B1g]	3044.46			2998	ν_{asym} (CH_3) + ν_{asym} (CH_2)
116	3045.36				A	ν_{asym} (CH_3) + ν_{asym} (CH_2)	[Au]	[B2g]	3045.18				ν_{asym} (CH_3) + ν_{asym} (CH_2)
117	3045.64				A	ν_{asym} (CH_3) + ν_{asym} (CH_2)	[B2g]	[Au]	3045.36				ν_{asym} (CH_3) + ν_{asym} (CH_2)
118	3046.29				A	ν_{asym} (CH_3) + ν_{asym} (CH_2)	[B3u]	[B3u]	3046.03				ν_{asym} (CH_3) + ν_{asym} (CH_2)
119	3065.96		2989		A	ν_{asym} (CH_3)	[B2u]	[B2u]	3065.95		2989		ν_{asym} (CH_3)
120	3066.38				A	ν_{asym} (CH_3)	[B1u]	[B1u]	3066.29				ν_{asym} (CH_3)
121	3066.51			3017	A	ν_{asym} (CH_3)	[B3g]	[B3g]	3066.50			3017	ν_{asym} (CH_3)
122	3066.90				A	ν_{asym} (CH_3)	[Ag]	[Ag]	3066.90				ν_{asym} (CH_3)
123	3072.24				A	ν_{asym} (CH_2) + ν_{asym} (CH_3)	[Au]	[Au]	3072.13				ν_{asym} (CH_2) + ν_{asym} (CH_3)
124	3074.23				A	ν_{asym} (CH_2) + ν_{asym} (CH_3)	[B1g]	[B3u]	3073.46				ν_{asym} (CH_2) + ν_{asym} (CH_3)
125	3074.43				A	ν_{asym} (CH_2) + ν_{asym} (CH_3)	[B3u]	[B1g]	3074.11				ν_{asym} (CH_2) + ν_{asym} (CH_3)
126	3076.02				A	ν_{asym} (CH_2) + ν_{asym} (CH_3)	[B2g]	[B2g]	3075.20				ν_{asym} (CH_2) + ν_{asym} (CH_3)

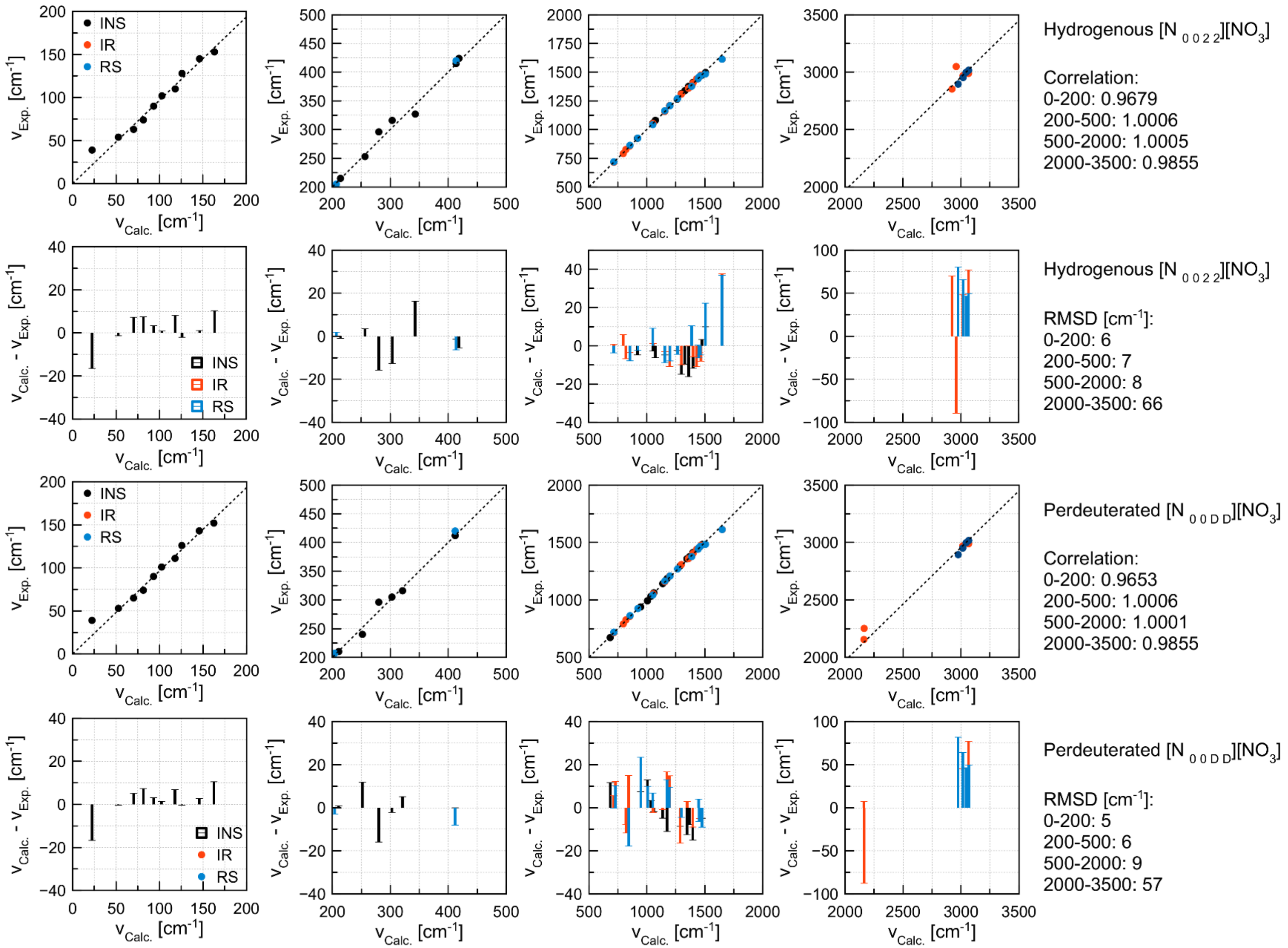

Figure S9 Quantitative analysis of the calculated vibrational frequencies (cm^{-1}) in hydrogenous and perdeuterated diethylammonium nitrate from fixed-cell calculations (CASTEP/PBE/NCPP/1050eV) against experimentally determined values with INS, IR, and Raman (RS) spectroscopies as divided into four different spectral regimes as indicated in the figures. The correlation between both sets of data has been presented along with the analysis of Root Mean Square Deviation (RMSD) errors.

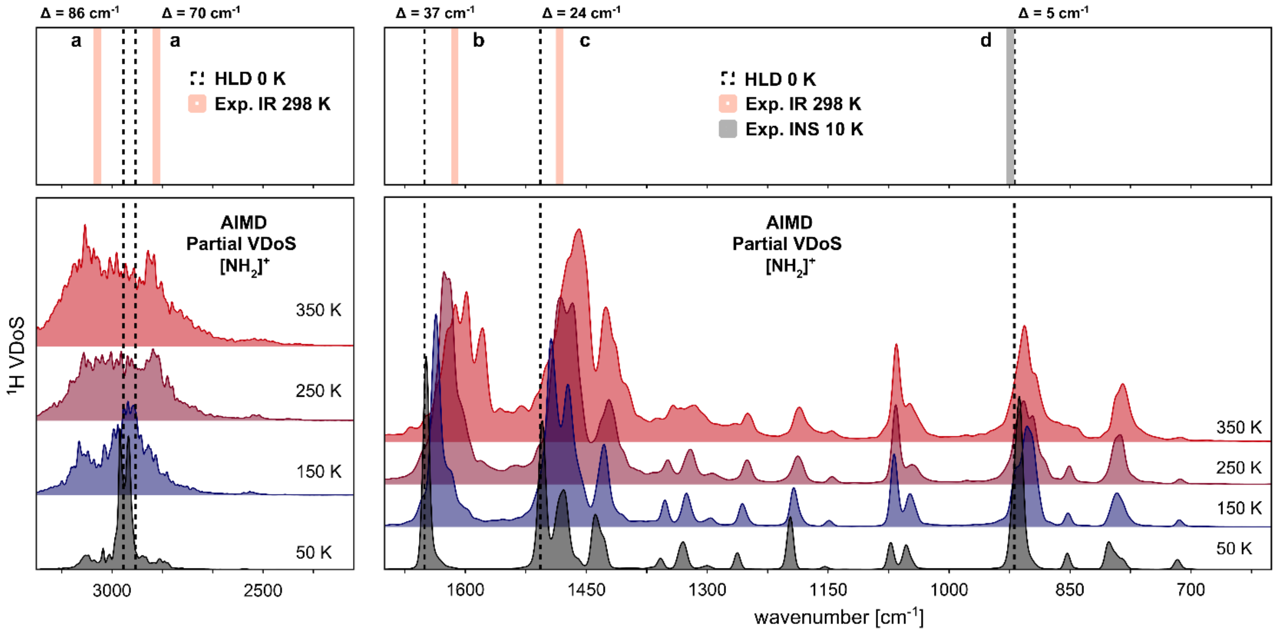

Figure S10 Hydrogen-projected VDoS from the [NH_2^+] fragments in hydrogeneous [$N_{0\,0\,2\,2}$][NO_3] at high (left) and intermediate (right) energy transfers, obtained from ab initio MD simulations (PBE/1050eV/hard-NCPP) in the microcanonical ensemble at selected temperatures. The top panel compares experimentally determined band positions with theoretical predictions within the harmonic approximation at zero temperature. The pink bands labelled a-d indicate shifts in band positions originating from anharmonicity.

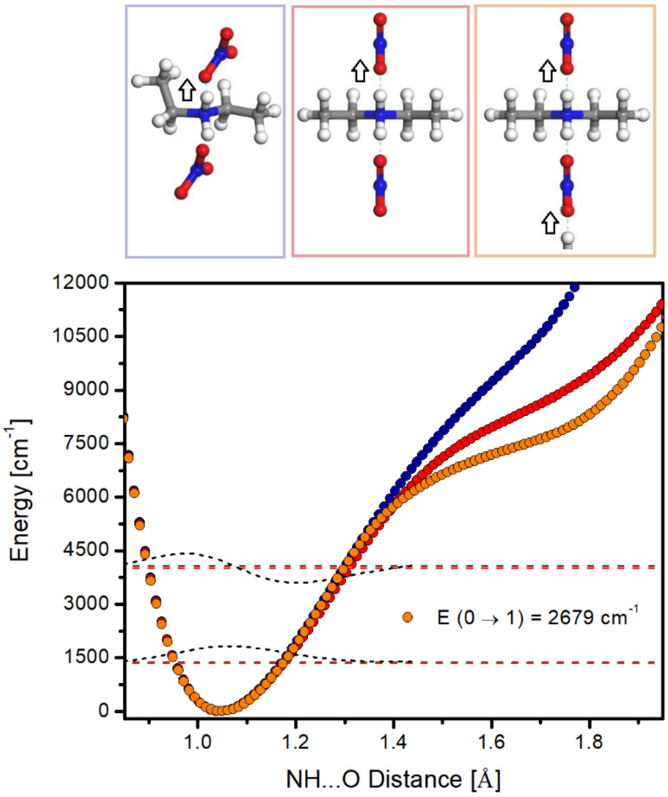

Figure S11 Potential-energy profiles of the hydrogen atom in the hydrogen bridges formed in crystalline hydrogeneous diethylammonium nitrate, [$N_{0\,0\,2\,2}$][NO_3]. The calculated profiles come from fixed-cell PBE/1050eV/hard-NCPPs calculations on the optimized low-temperature structures. These are color-coded according to the models given as cartoons at the top of the figure. All the calculations were performed on 2x2x2 supercell models by displacing the proton within a single H-bonded chain, as indicated by the arrows. The blue curve corresponds to a single proton displacement in the partially disordered system (left cartoon, see Fig. S12 and the 25% *gauche* model for more details). The red curve arises from single-proton displacements in the fully-ordered supercell (middle cartoon, see Fig. S12 and the 0% *gauche* structural model). The orange curve corresponds to collective proton displacements along the hydrogen-bond path (right cartoon). The 1-D Schrödinger equation for these energy profiles has been solved numerically and its eigenvalues are shown as dashed-colored horizontal lines for each model. The associated wavefunctions are shown for the case of the lowest-energy profile (orange curve, right cartoon).

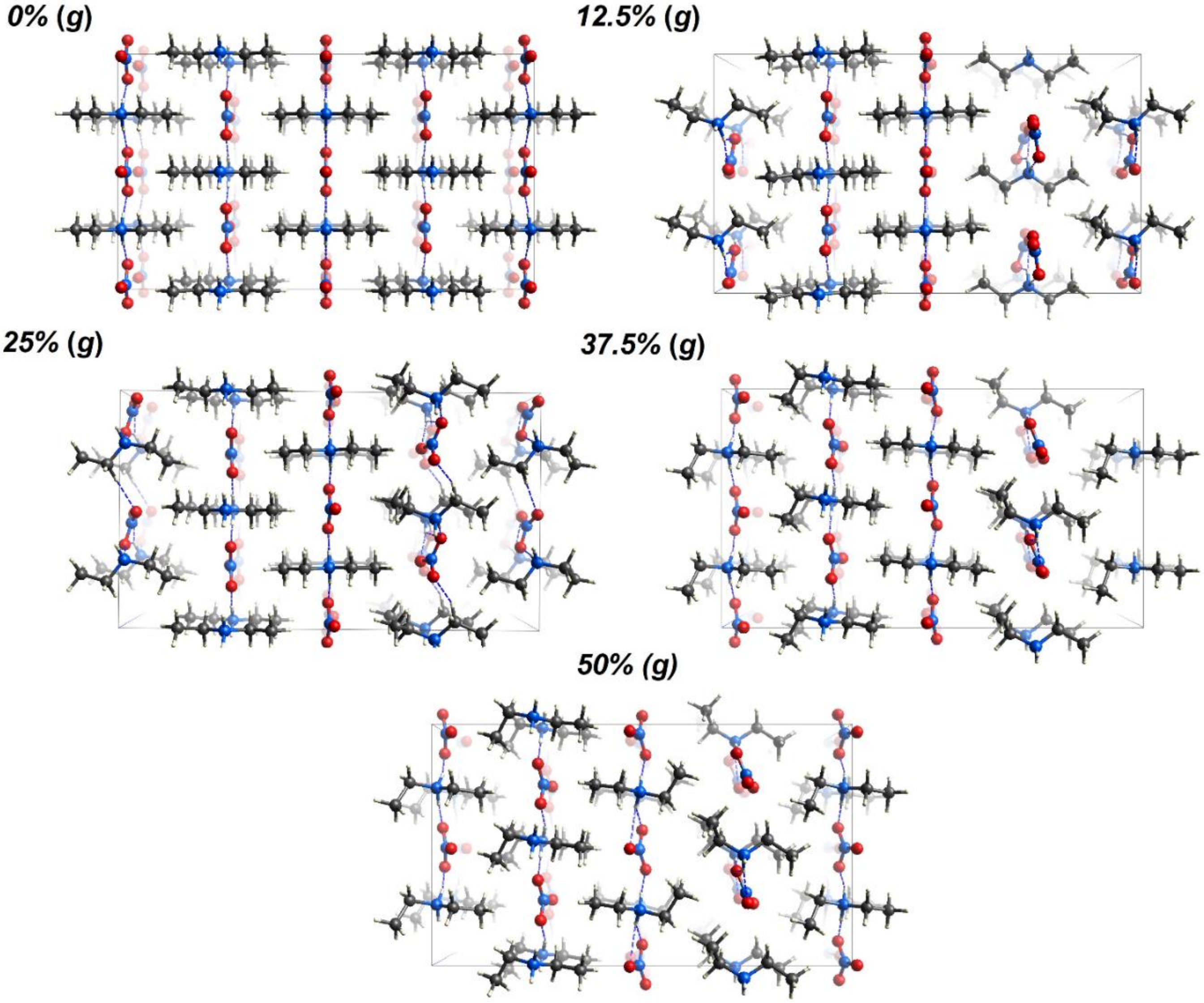

Figure S12 Extended supercell models to explore conformational disorder in $[N_{0\,0\,2\,2}][NO_3]$. All models consist of a total of 16 molecules and 32 diethyl fragments, and differ from each other in the percentage of diethyl-chain (*gauche or g)* conformers indicated in the figure.
